# ALS/FTD-associated mutation in cyclin F inhibits ER-Golgi trafficking, inducing ER stress, ERAD and Golgi fragmentation

**DOI:** 10.1038/s41598-023-46802-9

**Published:** 2023-11-22

**Authors:** Audrey M. G. Ragagnin, Vinod Sundaramoorthy, Fabiha Farzana, Shashi Gautam, Sayanthooran Saravanabavan, Zeinab Takalloo, Prachi Mehta, Dzung Do-Ha, Sonam Parakh, Sina Shadfar, Julie Hunter, Marta Vidal, Cyril J. Jagaraj, Mariana Brocardo, Anna Konopka, Shu Yang, Stephanie L. Rayner, Kelly L. Williams, Ian P. Blair, Roger S. Chung, Albert Lee, Lezanne Ooi, Julie D. Atkin

**Affiliations:** 1 Motor Neuron Disease Research Centre, Macquarie Medical School, Faculty of Medicine, Health and Human Sciences, Macquarie University, Sydney, NSW 2109 Australia; 2 Molecular Horizons and School of Chemistry and Molecular Bioscience, University of Wollongong, Northfields Avenue, Wollongong, NSW 2522 Australia; 3 La Trobe Institute for Molecular Science, La Trobe University, Bundoora, Melbourne, VIC 3086 Australia

**Keywords:** Cellular neuroscience, Molecular neuroscience, Neurological disorders

## Abstract

Amyotrophic lateral sclerosis (ALS) is a severely debilitating neurodegenerative condition that is part of the same disease spectrum as frontotemporal dementia (FTD). Mutations in the *CCNF* gene, encoding cyclin F, are present in both sporadic and familial ALS and FTD. However, the pathophysiological mechanisms underlying neurodegeneration remain unclear. Proper functioning of the endoplasmic reticulum (ER) and Golgi apparatus compartments is essential for normal physiological activities and to maintain cellular viability. Here, we demonstrate that ALS/FTD-associated variant cyclin F^S621G^ inhibits secretory protein transport from the ER to Golgi apparatus, by a mechanism involving dysregulation of COPII vesicles at ER exit sites. Consistent with this finding, cyclin F^S621G^ also induces fragmentation of the Golgi apparatus and activates ER stress, ER-associated degradation, and apoptosis. Induction of Golgi fragmentation and ER stress were confirmed with a second ALS/FTD variant cyclin F^S195R^, and in cortical primary neurons. Hence, this study provides novel insights into pathogenic mechanisms associated with ALS/FTD-variant cyclin F, involving perturbations to both secretory protein trafficking and ER-Golgi homeostasis.

## Introduction

Amyotrophic lateral sclerosis (ALS) is a fatal neurodegenerative disorder characterized by progressive degeneration of motor neurons, that overlaps with frontotemporal dementia (FTD)^1, 2^. Approximately 10% of cases are familial (FALS), which are clinically indistinguishable from sporadic ALS (SALS). Mutations in several genes, including *SOD1, TARDBP,* and *FUS*, are present in FALS, and we previously identified missense mutations in *CCNF* [NM_001761.3] (also called *FBXO1*) in patients with FALS/FTD^3^. One of the most common mutations in *CCNF* leads to substitution at position 621 (p.S621G). *CCNF* encodes cyclin F [NP_001752.2], a member of the cyclin protein family^4, 5^.

Whilst cyclins regulate the cell cycle^4, 6^, cyclin F does not activate cyclin-dependent kinases. However, it is the founding member of the F-box domain protein family^7^ that regulates formation of the E3 ubiquitin-protein ligase complex (SKP1-CUL1-F-Box-Protein)^7^. E3 ligases mediate ubiquitination and proteasomal degradation of target proteins via the ubiquitin proteasome system (UPS). Ubiquitination also regulates transport and localization of protein substrates^8^. ALS/FTD cyclin F^S621G^ disrupts Lys48-specific ubiquitination, leading to defects in autophagy^9, 10^. Overexpression of cyclin F^S621G^ in zebrafish results in abnormal motor axon morphology, which correlates with motor dysfunction^11^. Understanding ALS/FTD pathogenic mechanisms is important for designing effective therapeutic targets, but they remain poorly characterised for cyclin F-associated ALS/FTD.

Defects in protein homeostasis (proteostasis) are widely implicated in ALS/FTD^12, 13^ and the endoplasmic reticulum (ER) and Golgi apparatus compartments play important roles in this process. A major function of the ER is the production and transport of secretory/transmembrane proteins to the Golgi apparatus, from where they are sorted to their final destinations. We previously demonstrated that ALS-linked variants linked to ALS (SOD1, TDP-43, FUS, ubiquilin2) inhibit ER-Golgi transport^14, 15^. The first stage of ER-Golgi transport is budding of vesicles containing protein cargo from the ER containing the coat protein II (COPII) complex. COPII consists of Sec31 and Sec13, comprising the outer coat, and Sec23, Sec24 and Sar1 within the inner coat. After budding of COPII vesicles at specialised zones within the ER membrane, ‘ER exit sites’ (ERES)^16, 17^, tethering, docking and fusion to target membranes at the Golgi follows^18, 19^. Mono-ubiquitination of Sec31 drives assembly of large COPII coats^20^.

Dysfunction to ER homeostasis induces ER stress, which leads to the accumulation of misfolded/unfolded proteins within the ER, triggering the unfolded protein response (UPR). The UPR induces specific signalling pathways to reduce the load of misfolded proteins, and thus alleviate ER stress. This includes activation of ER-associated degradation (ERAD), whereby misfolded ER proteins are retro-translocated into the cytosol for degradation by the UPS, to ensure that only properly folded proteins are secreted^21^. Whilst initially protective, the UPR triggers apoptosis if prolonged or unresolved, hence ER stress is closely linked to cell survival.

Similarly, proper functioning of the Golgi apparatus is closely linked to cellular viability^22^, and its unique morphology, with tethered stacks of cisternae, is required for function. However, the Golgi apparatus can undergo disassembly and fragmentation into condensed, tubulovesicular punctate structures when its functions, including ER-Golgi trafficking, are compromised^22^, or during pathological conditions such as ALS^14, 23^.

Here we demonstrate that expression of ALS/FTD cyclin F^S621G^ in neuronal cells perturbs ER-Golgi homeostasis. ER-to-Golgi transport was inhibited in cyclin F^S621G^ expressing cells by a mechanism involving dysregulation of COPII vesicle formation at ERES. This was accompanied by activation of ER stress, Golgi fragmentation, ERAD and apoptosis. This study therefore reveals novel insights into pathogenic mechanisms induced by cyclin F variants in ALS/FTD, involving the ER-Golgi compartments.

## Methods

### Constructs

Expression constructs encoding wild-type (WT) and mutant S621G (and S195R for supplementary data) *CCNF* cDNA fused to C-terminal mCherry, were generated by subcloning *CCNF* cDNA into pmCherry-C1 vector (Addgene: https://www.addgene.org/32975/) as previously described^3^. The point mutations in *CCNF* (S621G or S195R) were introduced using a Q5® Site-Directed Mutagenesis kit, according to the manufacturer’s protocol (New England Biolabs E0554)^3^. Constructs encoding WT Venus or mutant single strand deglycosylation-dependant Venus (SS-ddVenus), where the C-terminus was fused to the unstable null Hong-Kong substrate (NHK-Venus; NHK-ddVenus)^24^, were a kind gift from Dr Jess E Grotzke (Department of Immunobiology, Yale University School of Medicine, New Haven, USA)^25^. The mutant vesicular stomatitis viral glycoprotein (*VSVG*^*ts045*^) fused to EGFP in the pEGFP-C1 vector was a kind gift from Dr Jennifer Lippincott-Schwartz (National Institutes of Health, Bethesda, USA).

### SH-SY5Y and HEK293T cell lines

Undifferentiated human neuroblastoma SH-SY5Y and human embryonic kidney HEK293T cell lines (ATCC) were cultured for 24 h to 80% confluence at 37 °C in a humidified atmosphere of 5% CO_2_ in Dulbecco’s modified Eagle’s medium (DMEM; Gibco), supplemented with 10% (v/v) heat-inactivated foetal bovine serum (Gibco). Authentication of cell line identities was confirmed via short tandem repeat profiling (Garvan Institute, Sydney). Cells were transfected for 48 h or 72 h with plasmids encoding pmCherry-C1 empty vector, WT or mCherry-tagged mutant S621G or S195R *CCNF*, or co-transfected with plasmids encoding pmCherryC1 empty vector, mCherry-tagged WT or mutant *CCNF* with plasmids encoding GFP-tagged VSVG^ts045^, or Venus/ddVenus using Lipofectamine™ 2000 (Invitrogen), following the manufacturer’s instructions. Neuronal SH-SY5Y cells were selected for immunocytochemistry studies, whereas HEK293T cells were used primarily for Western blotting studies because of their high transfection efficiency.

### Mouse primary cortical neurons

Primary neurons were harvested from the cortex of C57BL/6 mouse embryos at embryonic day 16–18. The culture of primary neurons was performed as described previously^26^. Briefly, cortical tissue was dissected, cut into pieces under sterile conditions in Hanks’ Balanced Salt solution (Gibco) and digested in 10 units/ml papain (Sigma) in 0.2 mg/ml L-cysteine, 1 mM CaCl_2_ and 0.5 mM EDTA (pH 8) in DMEM (Gibco) for 10 min at 37 °C. Cells were subsequently dissociated, resuspended in platting medium (Neurobasal medium [Gibco] supplemented with 10% (v/v) heat-inactivated foetal bovine serum [Gibco], 2% (v/v) B-27 supplement [Gibco], 1% (v/v) GlutaMAX™ [Gibco] and 100 µg/ml penicillin–streptomycin) and seeded for 12 h on 15 mm glass coverslips previously coated overnight with 0.1 mg/ml poly-D-lysine (Sigma). Cells were then incubated in neuronal medium (Neurobasal medium [Gibco] supplemented with 2% (v/v) B-27 supplement [Gibco], 1% (v/v) GlutaMAX™ [Gibco] and 100 µg/ml penicillin–streptomycin) at 37 °C in a humidified atmosphere of 5% CO_2_. Half of the medium was changed every three days. After 5 days in vitro, neurons were transfected with constructs encoding mCherry, WT or mutant S621G or S195R *CCNF* using Lipofectamine™ 2000 (Invitrogen) following the manufacturer’s instructions. Primary neurons were then incubated for 48 h before fixation in 4% paraformaldehyde (PFA) in 0.1 M PBS.

### Differentiation of induced pluripotent stem cells (iPSCs) to spinal motor neurons

All experimental protocols were approved by the University of Wollongong Research Ethics Committee (HE13/272). The methods were carried out in accordance with the guidelines as set out in the National Statement on Ethical Conduct in Research Involving Humans and informed consent was obtained from all donors. Generation and characterisation of human feeder-free iPSCs from an ALS patient carrying the cyclin F^S621G^ mutation was as previously described^27^. The patient-derived iPSCs and CRISPR-corrected isogenic control were maintained on Matrigel coated tissue culture plates in mTeSR1 at 37 °C, 5% CO_2_, 3% O_2_ in a humidified incubator prior to differentiation to motor neurons. Motor neuron differentiation was based on a protocol previously described^28^, with slight alterations. Briefly, neural induction was initiated under SMAD inhibition with LDN193189 (1 µM). Floating embryoid bodies (EBs) were generated by lifting iPSC colonies and culturing these in suspension with neural induction (Ni) media (DMEM/F12, with 0.4% (v/v) B27, 1% (v/v) N2, 1% (v/v) non-essential amino acids and 1% (v/v) GlutaMAX) supplemented with SB431542 (2 µM) and CHIR99021 (3 µM) for 5 days. Floating EBs were plated on Matrigel coated tissue culture plates in Ni media with SB431542, CHIR99021 and DMH-1 (2 µM) to generate neural precursor cells (NPCs). After 5 days, NPCs were primed to caudal and ventral identity with the addition of retinoic acid (RA; 0.1 µM) and purmorphamine (Pur, 0.5 µM) resulting in anterior NPCs (aNPCs). The aNPCs were matured in aNPC maturation media (BrainPhys with 0.5% B-27 (v/v), 1% N2 (v/v), 1% (v/v) non-essential amino acids) supplemented with SB431542, CHIR99021, DMH-1, RA and Pur. For motor neuron differentiation and maturation, aNPCs were replated on Matrigel coated tissues plates at a density of 40 000 cells/cm^2^ in motor neuron maturation medium (BrainPhys with 1% (v/v) B27, 1% (v/v) N2, 1% (v/v) non-essential amino acids) supplemented with BDNF, GDNF, IGF-1 (at 10 ng/mL), 500 ng/mL cAMP (500 ng/mL) L-ascorbic acid (200 ng/ml), RA (0.5 µM), Pur (0.1 µM) and γ-secretase inhibitor DAPT (10 µM). Motor neuron cultures were maintained for 12 days prior to protein harvest.

*Cell lysis* iPSC-derived motor neurons were harvested after 12 days of maturation for western blot analysis. Motor neurons were washed with PBS and lysed with RIPA buffer (50 mM Tris-HCl pH 7.4, 150 mM NaCl, 0.1% SDS and 1% protease inhibitors). Cell lysate was subsequently centrifuged at 16,000 rcf for 10 min at 4 °C and the supernatant (the RIPA soluble protein fraction) was collected.

### Immunocytochemistry

SH-SY5Y and HEK293T cells grown on 13 mm coverslips were washed in 0.1 M PBS (pH 7.2) and fixed in 4% PFA in PBS for 10 min. After 3 washes in PBS, cells were permeabilised in 0.1% (v/v) Triton X-100 in PBS for 5 min and the non-specific background staining was blocked, using 3% (w/v) bovine serum albumin (BSA) in PBS for 45 min at room temperature with gentle rocking. Cells were then incubated overnight at 4 °C with primary antibodies diluted in 1% (w/v) BSA in PBS: polyclonal rabbit anti-calnexin (1:100; Abcam 22,595) or monoclonal mouse anti-CHOP (1:50; Santa Cruz sc7351), anti-XBP1 M-186 (1:25; Santa Cruz sc-7160), anti-FLAG (1:500; Sigma F3165), anti-Sec31A (1:100; BD Biosciences 612,351), or anti-GM130 (1:50; BD Transduction 610,823). After rinsing, cells were incubated for 1 h at room temperature with gentle rocking and the appropriate secondary antibodies (diluted 1:250 in PBS) coupled to either Alexa 647 (Life Technology), Alexa 594 (Molecular Probes) or Alexa 488 (Life Technology) were added. Cells were then washed as above and treated with 0.5 µg/ml Hoechst 33,342 reagent (Sigma). After 3 washes in PBS, coverslips were mounted onto slides in fluorescent mounting medium (Dako) and cells were photographed with 20x/na = 0.8, 40x/na = 1.3, 63x/na = 1.4 or 100x/na = 1.46 objectives on a Zeiss LSM 880 inverted confocal laser-scanning microscope, equipped with a LSM-TPMT camera (Zeiss). Additional low-resolution images were acquired with 20x/na = 0.5, 40x/na = 0.75, 63x/na = 1.4 or 100x/na = 1.46 objectives on an AxioImager Z2 fluorescent microscope (Zeiss) equipped with a monochrome AxioCamHRm digital CCD camera (Zeiss).

### VSVG^ts045^ transport assay

SH-SY5Y cells co-expressing cyclin F and VSVG^ts045^ were incubated overnight at 40 °C to accumulate VSVG^ts045^ in the ER. Cycloheximide (Sigma) diluted 20 µg/ml in DMEM was then added and the cells were incubated at 32 °C for 30 min to allow VSVG^ts045^ to traffic to the Golgi. After one wash in PBS, samples were fixed in 4% PFA in 0.1 M PBS (pH 7.2) for 10 min and processed for immunocytochemistry as described above. At least 20 cells expressing both cyclin F and VSVG^ts045^ were examined in each group.

### *In vitro* budding assay

A modified in vitro assay was used to analyse ER vesicle budding^14, 29^. Briefly, HEK293T cells co-transfected with constructs encoding cyclin F and VSVG^ts045^ were incubated overnight at 40 °C to accumulate VSVG^ts045^ in the ER. Cells were then washed in PBS, resuspended in 5/90 buffer (50 mM HEPES and 90 mM potassium acetate in demineralized water) and incubated with rat liver cytosol (Thermofisher) and an energy regenerating system (50 mM creatine phosphate, 0.2 mg/ml creatine phosphokinase and 1 mM ATP) at 32 °C for 30 min. Identical samples were incubated at 4 °C to monitor non-specific ER fragmentation. The cells were removed by low-speed centrifugation at 4000*g* for 1 min at 4 °C, followed by 15000*g* for 1 min, and budded vesicles were recovered by centrifugation at 100,000*g* for 1 h at 4 °C from the resulting supernatant. The levels of VSVG^ts045^ cargo in the budded vesicle fractions were quantified by Western blotting using anti-VSVG (1:1000; Sigma V4888) and anti-COPII (Sec23; 1:500; Pierce, Rockford, IL, PA1-069A) antibodies. The relative intensity of VSVG and COPII/Sec23 to β-actin was normalised to untreated cells.

### Quantification of Sec31-positive clusters

Images acquired by confocal microscopy under × 100 objective were used for analysis of Sec31-positive clusters in whole cells. Individual cells were outlined by the freehand selection tool in ImageJ, converted to RGB images and colour threshold level set to 40–255. The ‘Analyze particles’ feature of ImageJ was then used to obtain diameter of selected clusters. Eight to ten individual cells per group were analyzed. A size distribution curve was plotted for 100 nm bins using the pivot table function in Microsoft Excel.

### Western blotting

HEK293T cells transfected with constructs encoding cyclin F for 48 h were washed in PBS, lysed in 50 mM Tris-HCl (pH 7.4), 150 mM NaCl, 0.1% (w/v) SDS, 1% protease inhibitor cocktail (Sigma) and 1% phosphatase inhibitor cocktail (Sigma). The supernatant was then cleared by centrifugation at 12,000*g* for 10 min at 4 °C. The total amount of protein in each sample was quantified using a Pierce BCA Protein Assay Kit (Thermo Fisher Sci.), following the manufacturer’s instructions. Proteins in the medium fraction were concentrated using Amicon Ultra (0.5 ml) columns (Merck). Proteins (10–20 µg) were separated on 7.5%, 4–15% or 4–20% gels (BioRad) and either stained with a Silver Stain kit (Thermo Fisher) or transferred onto nitrocellulose membranes according to the manufacturer’s instructions (BioRad). Blots were pre-incubated in blocking solution containing 5% (w/v) skim milk in Tris-buffered saline, followed by incubation overnight at 4 °C in primary antibodies diluted in blocking solution: polyclonal rabbit anti-cyclin F (1:500; Santa Cruz sc952), anti-VSVG (1:1000; Sigma V4888), anti-COPII/Sec23 (1:500; Pierce, Rockford, IL, PA1-069A), anti-Xbp1 (1:500, Santa Cruz, SC-8015), monoclonal mouse anti-Sec31A (1:500; BD Biosciences 612,351), anti-GAPDH (1:10,000, Abclonal, A19056 [iPSC] or 1:5000, ProteinTech, 60,004-I-Ig [HEK]), anti-calnexin (1:1000, Abcam, ab22595) or anti-β-actin (1:4000; Sigma A5441 [AC-15]) antibodies. After rinsing, blots were incubated in peroxidase-conjugated secondary antibodies (1:2000 [anti-rabbit] or 1:3000 [anti-mouse], Millipore) for one hour at room temperature or processed using fluorescent antibodies (Li-Cor Biosciences) following the manufacturer’s instructions. Immunoreactivity was revealed using the Clarity™ ECL Western Blotting Substrate (BioRad) and images were obtained either with the BioRad ChemiDoc MP system using Image Lab™ software (BioRad) or with Odyssey^®^ CLx and Image Studio software (Li-Cor Biosciences). The intensity of each band relative to β-actin was quantified using ImageJ.

### ERAD assay

SH-SY5Y cells co-transfected with constructs encoding cyclin F and either NHK-Venus, NHK-ddVenus or SS-ddVenus for 48 h, were fixed in 4% PFA in 0.1 M PBS, pH 7.2, and mounted as above. Images were acquired using an Axio Imager Z2 fluorescent microscope at 20x/na = 0.8 magnification. At least 100 cells expressing cyclin F and Venus/ddVenus were scored as the percentage of NHK-Venus, NHK-ddVenus or SS-Venus fluorescent cells from three different experiments.

### Subcellular fractionation to produce ER-rich fractions

HEK293T cells expressing cyclin F for 48 h were resuspended in 500 μl fractionation buffer, containing 20 mM HEPES (pH 7.5), 10 mM KCl, 1.5 mM MgCl_2_, 1 mM EDTA, 1 mM DTT and 0.1% protease and phosphatase inhibitor cocktail (Roche). Cells were passed 10 times through a 27 G needle and incubated at 4 °C for 20 min. The cell lysate was then centrifuged at 720*g* for 5 min and the supernatants, containing the cytoplasmic, membrane and mitochondrial fractions, were centrifuged at 10,000*g* for 5 min to pellet the mitochondrial fraction. The supernatant was then centrifuged at 100,000*g* for 1 h to pellet the membrane fraction and washed again by adding 200 μl of fractionation buffer, following resuspension by pipetting and passing through a 25-gauge needle. Following centrifugation again for 1 h at 100,000*g*, the pellet was resuspended in 100 μl RIPA buffer with 0.1% SDS and retained as the ER fraction.

### Co-localization analysis between calnexin and cyclin F

Mander’s coefficient was calculated for each cell to determine the degree of colocalisation (where 0 indicated no colocalisation and 1 indicated total colocalisation) of VSVG^ts045^ (50 + cells per group) or cyclin F (20 + cells per group) with either calnexin or GM130, using the JaCoP plugin^30^ in ImageJ (http://rsbweb.nih.gov/ij/index.html). All experiments were performed in triplicate.

### Quantitative analysis of cells displaying ER stress

The percentage of cells displaying nuclear immunoreactivity to CHOP or XBP1 as specific markers was quantified from 30 + primary neurons per group and at least 100 + HEK293T cells per group expressing cyclin F from n ≥ 3 independent experiments. Apoptotic cells identified by their condensed or fragmented nucleus^31^ were excluded from the analysis.

### Quantitative analysis of cells with Golgi fragmentation

The percentage of cells expressing cyclin F with Golgi fragmentation was quantified from 10 to 30 primary neurons per group and from at least 50 + SH-SY5Y or 100 + HEK293T cells per group from n = 3 independent experiments. Only cells where the Golgi structure was clearly visible were analysed. Normal Golgi morphology was evident by the presence of continuous stacked membranous vesicles. In contrast, fragmented Golgi were detected by the presence of disconnected elements and tubular-vesicular clusters. The Golgi was considered fragmented when at least 5 fragments were clearly visible. The area covered by the Golgi fragments was also calculated using ImageJ. Apoptotic cells identified by their condensed or fragmented nucleus^31^ were excluded from the analysis. All analyses were performed blind.

### Quantitative analysis of apoptotic nuclei

Apoptotic nuclei were defined as condensed when they were under 5 µm in diameter or fragmented (multiple condensed Hoechst-positive structures in one cell), following previous methods^31^. The percentage of apoptotic cells was quantified from 10 to 30 primary neurons per group or from at least 100 + HEK293T cells per group expressing cyclin F from n ≥ 3 independent experiments. Cells undergoing division were excluded from analysis.

### Sytox Blue staining

HEK293T transfected with cyclin F constructs for 72 h were harvested by adding trypsin for 1 min at room temperature. The cells were then collected in PBS, centrifuged at 1200 rpm for 5 min and resuspended in 200 µl of buffer containing 10 mM HEPES, 140 mM NaCl, 2.5 mM CaCl_2_, pH 7.4. The cell suspension was treated with 1 µM Sytox Blue nucleic acid stain (Invitrogen) for 10 min at room temperature in the dark. The cell suspension was then analysed for SYTOX blue positive cells after gating for cells positive for mCherry fluorescence, using a BD FACS Canto™ II flow cytometer (BD Biosciences).

### Statistics

Data are presented as mean value ± standard error of the mean (SEM). Statistical comparisons between group means were performed using GraphPad Prism 6 software (Graph Pad software, Inc.). One-way or two-way ANOVA followed by post hoc Tukey test for multiple comparisons was used when justified. The significance threshold was set at *p* = 0.05. Size distribution curves for ERES clusters were plotted for 10 nm bins in Airyscan resolved images and 100 nm bins for whole cell images. Binning was performed using the pivot table function in Microsoft Excel.

### Ethical approval

All husbandry and experimental procedures were performed in compliance with the Animal Ethics Committee, Macquarie University, NSW, Australia (ARA 2017/030) and the Internal Biosafety Committee, Macquarie University (NLRD 5,201,401,007 and 5974–52,019,597,412,350).

## Results

### ALS/FTD cyclin F^S621G^ inhibits ER-Golgi transport

**Figure 1:**
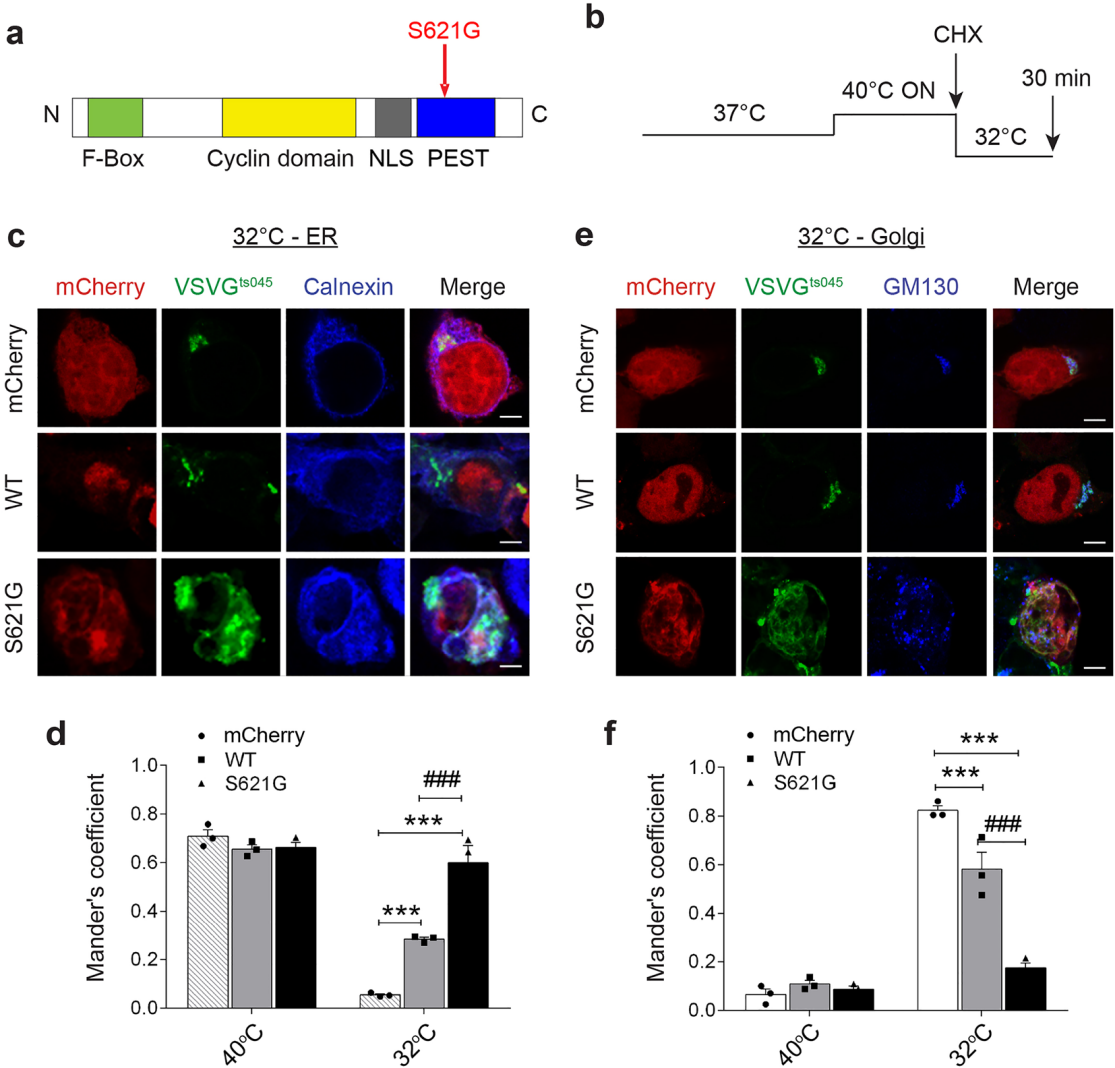
ALS/FTD cyclin F^S621G^ inhibits ER to Golgi trafficking. (**a**) Schematic diagram illustrating domain structure of cyclin F showing the location of the p.S621G mutation in the PEST domain. NLS, nuclear localisation signal. (**b**) Experimental paradigm: cells expressing VSVG^ts045^ for 24 h are incubated at 40°C overnight (ON) to misfold VSVG^ts045^, leading to its accumulation in the ER. Cells are then incubated for 30 min in cycloheximide (CHX) to inhibit further VSVG^ts045^ protein synthesis to synchronously release it from the ER before transport to the Golgi. When cells are placed at the permissive temperature (32 °C), VSVG^ts045^ refolds and transits to the Golgi apparatus within 30 min. (**c**) Confocal microscopy images following immunocytochemistry for calnexin (as an ER marker) in SH-SY5Y cells co-expressing GFP-tagged VSVG^ts045^ and either mCherry only (mCherry), cyclin F ^WT-^mCherry (WT) or variant cyclin F^S621G^ mCherry (S621G), after incubation at 40 °C overnight and 32 °C for 30 min as in (**b**). Scale bar = 5 µm. (**d**) The degree of co-localisation of VSVG^ts045^ with calnexin from the images in (**c**) was quantified using Mander’s coefficient. (**e**) Confocal microscopy images following immunocytochemistry for GM130 (as a Golgi marker) in SH-SY5Y cells co-expressing GFP-tagged VSVG^ts045^ and either mCherry only (mCherry), cyclin F ^WT-^mCherry (WT) or cyclin F^S621G^ mCherry (S621G), after incubation at 40 °C overnight and 32 °C for 30 min as in (**b**). (**f**) The degree of colocalisation of VSVG^ts045^ with GM130 from the images in (**c**) was quantified using Mander’s coefficient. (**d**, **f**) Graphs represent mean ± SEM. Symbols represent 3 independent experiments, two-way ANOVA (factors: “temperature” and “genotype”) followed by post-hoc Tukey test for multiple comparisons; ***p < 0.001 vs cells expressing mCherry only, ^###^*p* < 0.001 vs cells expressing cyclin F^WT^. After switching to the permissive temperature (32 °C), VSVG^ts045^ is retained in the ER and less is transported to the Golgi apparatus, in cells expressing cyclin F^S621G^ compared to both WT and mCherry only cells. However at 40 °C, VSVG^ts045^ is retained in the ER in all groups, demonstrating the specificity of the assay.

We expressed p.S621G ALS/FTD-associated cyclin F (Fig. 1a)^3^, tagged with mCherry, in two cell lines, SH-SY5Y cells and HEK293T. We previously confirmed similar expression of cyclin F^S621G^ to cyclin F^WT^ in two cell lines, including HEK293T^11^. SH-SY5Y cells were used in immunocytochemistry studies only, due to the low transfection efficiency of cyclin F in this cell line, where individual cells were analysed. Only transfected cells were included so that outcomes were not dependent on transfection efficiency.

We first examined if cyclin F^S621G^ inhibits ER-Golgi trafficking following widely used methods^14, 15^, using a temperature sensitive mutant of vesicular stomatitis viral glycoprotein (VSVG^ts045^)^14, 32–34^ as a reporter^35, 36^ (Fig. 1b). Cells co-expressing GFP-tagged VSVG^ts045^ and mCherry-tagged cyclin F were incubated overnight at 40 °C to accumulate misfolded VSVG^ts045^ in the ER. They were then incubated for 30 min at 32 °C to refold VSVG^ts045^ with cycloheximide (CHX) to inhibit protein synthesis, so that VSVG^ts045^ is released synchronously from the ER, before transport to the Golgi apparatus. VSVG^ts045^ transport was examined by immunocytochemistry using anti-calnexin (Fig. 1c) or anti-GM130 (Fig. 1e) antibodies to label the ER and Golgi compartments, respectively. Mander’s coefficient was used to quantify the degree of colocalization between fluorophores, representing the extent of overlap between images. This was quantified in a range from 0, indicating no co-localisation to 1, representing high co-localisation.

VSVG^ts045^ co-localised significantly more with calnexin in cyclin F^S621G^ expressing cells (0.6 ± 0.03), after incubation at the permissive temperature (32 °C), compared to control cells expressing either cyclin F^WT^ (0.3 ± 0.03) or mCherry only (0.06 ± 0.009) (Fig. 1d). Hence more VSVG^ts045^ was retained in the ER in cyclin F^S621G^ expressing cells compared to controls. Consistent with these findings, in cells expressing cyclin F^S621G^ significantly less VSVG^ts045^ colocalised with GM130 (0.2 ± 0.02) compared to controls expressing mCherry only (0.8 ± 0.02) or cyclin F^WT^ (0.6 ± 0.03) (Fig. 1f). Hence, VSVG^ts045^ extensively colocalised with GM130 in control cells, indicating efficient ER-Golgi transport, but not in cyclin F^S621G^ cells. Additional controls from each group were also examined whereby cells remained at 40 °C incubation for 30 min after CHX treatment, instead of switching to the permissive temperature (32 °C) (Supplementary Fig. 1). As expected, VSVG^ts045^ was retained within the ER for each group at 40 °C and there were no differences detected in either ER or Golgi localisation (Fig. 1d, f). Inhibition of ER–Golgi transport was not caused by non-specific over-expression of protein, because efficient VSVG^ts045^ transport was detected in cells expressing mCherry (27 kDa) only, where little retention in the ER was observed (Fig. 1d, f). Moreover, we previously established that transport of VSVG^ts045^ was not inhibited in cells expressing wildtype versions of SOD1, TDP-43, FUS, or UBQLN2 or GFP only^14, 15^. Similarly, we also previously confirmed that a mutant protein unrelated to ALS and neurodegeneration did not inhibit ER-Golgi transport of VSVG^ts045^ in this assay^14^: R311K Nck adaptor protein 2^37^. However, we detected a small, but significant, inhibition of transport in cells expressing cyclin F^WT^ compared to mCherry only, but this was significantly less than cyclin F^S621G^. Therefore, in summary, more VSVG^ts045^ is retained in the ER and less is transported to the Golgi in cells expressing ALS/FTD-variant cyclin F compared to controls expressing cyclin F^WT^ cyclin F or mCherry only, indicating perturbation of ER-Golgi transport.

### ALS/FTD-associated cyclin F^S621G^ inhibits COPII vesicular ER budding

**Figure 2:**
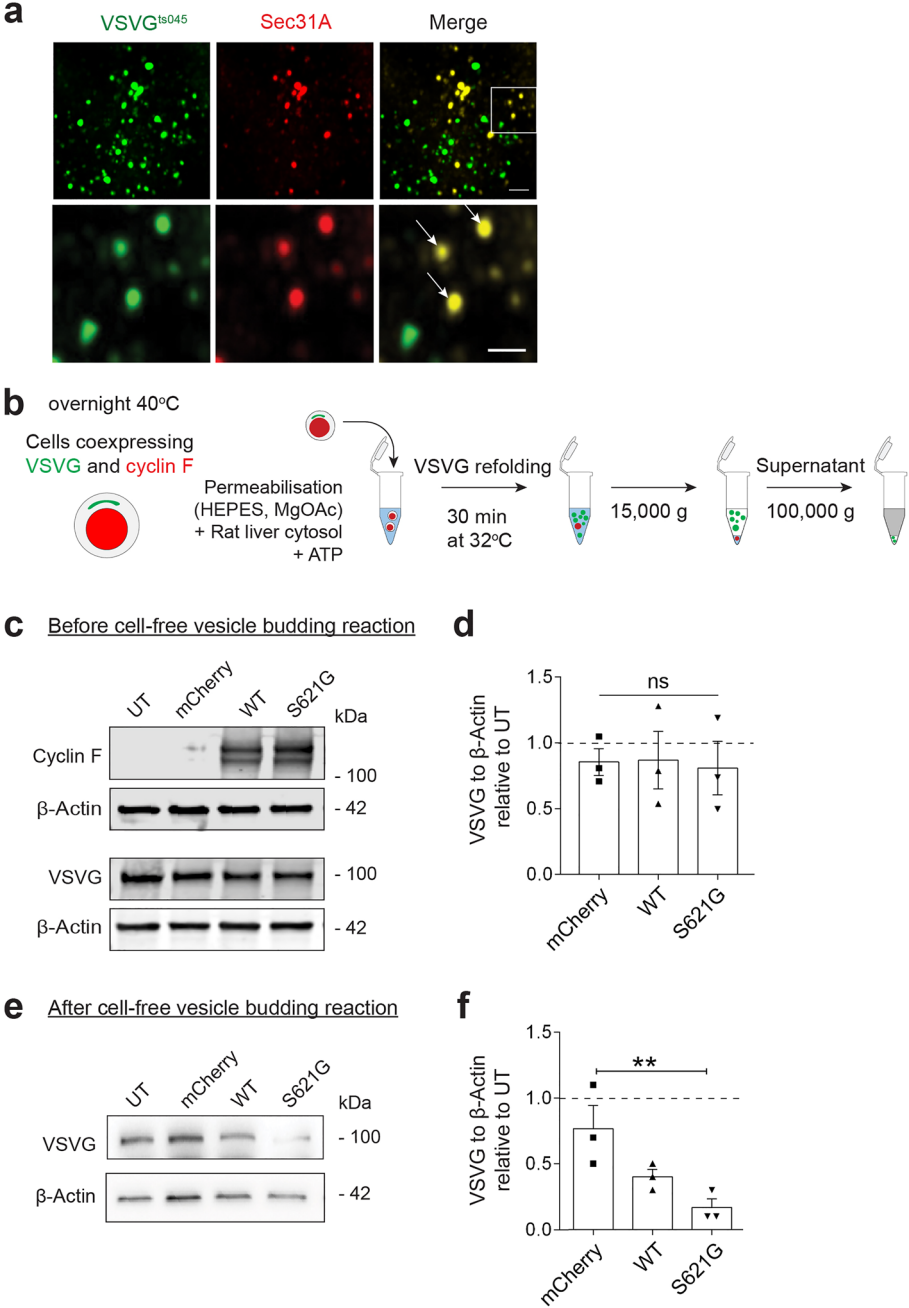
ALS/FTD cyclin F^S621G^ inhibits the load of protein cargo (VSVG^ts045^) into COPII vesicles. (**a**) Immunocytochemistry for Sec31 in SH-SY5Y cells expressing GFP-tagged VSVG^ts045^, after incubation at 40 °C overnight and 32 °C for 30 min. *Upper panel:* Low magnification images of cells immunostained for Sec31; region enlarged in the lower panel is indicated by box shown in the inset. *Lower panel:* higher magnification image of inset, illustrating the presence of VSVG^ts045^ in Sec31-positive vesicles. Scale bar = 1 *µ*m [*upper panel*] or 0.5 *µ*m [*lower panel*]. VSVG^ts045^ co-localises with Sec31 (arrows), and hence COPII vesicles. (**b**) Schematic diagram depicting the experimental procedure used for cell-free ER vesicle budding reactions. The same number and volume of HEK293T cells for each group were co-transfected with VSVG^ts045^ and either mCherry only, cyclin F-mCherry (WT or variant), or untransfected cells as a control. VSVG^ts045^ was trapped in the ER by incubating cells overnight at 40 °C and ER-vesicle budding was reconstituted in vitro in perforated cells expressing mCherry only or mCherry-tagged cyclin F proteins by incubating cells at 32 °C with rat liver cytosol as a source of ER membranes and an ATP regenerating system. Budded vesicles were collected by centrifugation. (**c**) Western blotting of cyclin F and VSVG^ts045^ in lysates from untransfected cells (UT) or cells expressing mCherry only, cyclin F^WT^-mCherry (WT), or variant cyclin F^S621G^-mCherry (S621G), before budding was performed and ER-derived vesicles were isolated. β-actin was used as a loading control for protein expression. (**d**) Relative intensity of VSVG^ts045^ to β-actin (normalized to control, untransfected cells: a line parallel to the x axis set at 1) in (**c**). Mean ± SEM. Symbols represent independent experiments, one-way ANOVA followed by post-hoc Tukey test for multiple comparisons, ns, non-significant. No differences in expression of VSVG^ts045^ between groups were detected. (**e**) Western blotting for cyclin F and VSVG^ts045^ of ER-derived vesicle preparations isolated from untransfected cells (UT) and cells expressing cyclin F^WT^-mCherry (WT), or cyclin F^S621G^-mCherry (S621G), after vesicle budding was performed. β-actin was used as a loading control for protein expression. (**f**) Relative intensity of VSVG^ts045^ in blots in (**e**) normalised to β-actin and control, untransfected cells (a line parallel to the x axis set at 1). Mean ± SEM. Symbols represent independent experiments, one-way ANOVA followed by a post-hoc Tukey test for multiple comparisons,   **p < 0.01 *vs* cells expressing mCherry or untransfected cells (UT).

ER-Golgi trafficking is initiated when COPII vesicles (containing protein cargo) bud from the ER membranes^18, 19^. We next examined whether this initial stage of transport was inhibited in cells co-expressing cyclin F and VSVG^ts045^ as a cargo^14, 32–34^ (Fig. 2). Sec31 and Sec23 were employed as specific markers of newly-budded, ER-derived COPII vesicles at ERES.

First, to confirm that VSVG^ts045^ is transported from ER-to-Golgi in COPII vesicles, cells expressing GFP-tagged VSVG^ts045^ were incubated overnight at 40 °C to accumulate misfolded VSVG^ts045^ in the ER, followed by 30 min incubation at the permissive temperature (32 °C) to allow VSVG^ts045^ to transport to the Golgi. Immunocytochemistry for Sec31 (as outer coat marker) revealed that VSVG^ts045^ was associated with Sec31-positive vesicles (Fig. 2a), consistent with its packaging into COPII vesicles exported from the ER.

We next used a modified in vitro assay^29^ to examine vesicle budding from the ER (Fig. 2b). This assay examines whether VSVG^ts045^-GFP is associated with membranes that form following ER retention, including those from COPII vesicles, the ER-Golgi intermediate compartment (ERGIC) and intra/post Golgi carriers. It therefore aims to reconstitute ER vesicle budding using semi-intact cells (as a source of ER), rat liver cytosol (as a source of soluble COPII) and an ATP regeneration system (Fig. 2b). ER-derived COPII vesicles are released into the buffer during incubation^29, 38^, given that the integrity of the ER is preserved under these conditions^29, 39, 40^. Semi-intact HEK293T cells co-expressing cyclin F and VSVG^ts045^ for 24 h were incubated at 40 °C overnight to retain VSVG^ts045^ in the ER, then incubated at 32 °C to allow VSVG^ts045^ incorporation into COPII vesicles. The light membranes, including ER-derived vesicles, were then recovered by cellular fractionation (Fig. 2b).

Control experiments revealed that if cells were incubated at 40 °C rather than the permissive temperature, VSVG^ts045^ was not incorporated into COPII vesicles after the budding reaction, confirming that it is retained in the ER at 40 °C (Supplementary Fig. 2a). VSVG^ts045^ was also examined in control lysates, sampled before the budding reaction, to examine its expression in the different cell populations. However, no significant differences in VSVG^ts045^ were detected between all groups (Fig. 2 c, d). Similarly, no significant differences in expression of Sec23, an inner COPII coat marker, were present amongst all cell lysates before the budding reaction (Supplementary Fig. 2b,c). Hence there are similar levels of protein expression in all the different populations, ruling this out as a possible confounding factor.

VSVG^ts045^ was then quantitated in budded vesicular preparations by Western blotting for VSVG (Fig. 2d,e). Significantly less VSVG^ts045^ was present in cells expressing cyclin F^S621G^ compared to untransfected cells (3.4-fold) and cells expressing mCherry only (3.2-fold) (Fig. 2f). COPII was also assessed in fractions after budding using Sec23 as a marker of the vesicular interior (Supplementary Fig. 2d). Significantly less Sec23 was present in vesicles obtained from cells expressing cyclin F^S621G^ compared to untransfected cells and mCherry only expressing cells (3-4 fold) (Supplementary Fig. 2e), consistent with less COPII vesicular transport.

Hence, less COPII and protein cargo (VSVG^ts045^) are present in ER-derived vesicles prepared from cells expressing cyclin F^S621G^ compared to controls. This suggests that the first step of ER-Golgi trafficking, vesicle budding from the ER, is perturbed by cyclinF^S621G^ in ALS/FTD.

### ERES are perturbed in cells expressing ALS/FTD-associated cyclin F^S621G^

**Figure 3:**
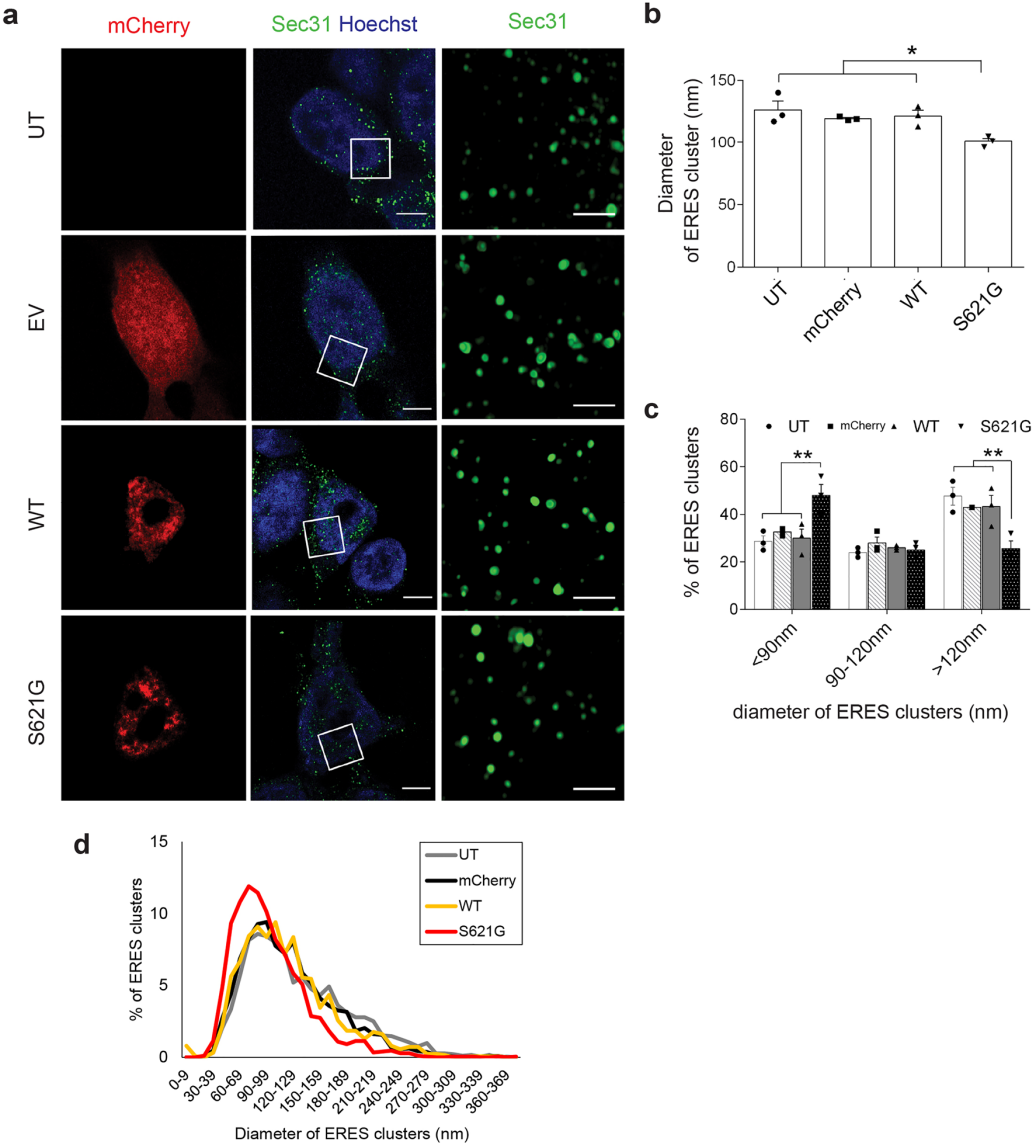
ALS/FTD cyclin F^S621G^ perturbs ER-exit sites. (**a**) Fluorescent confocal airyscan microscopy images of HEK293T cells following immunocytochemistry for Sec31 and Hoechst staining in untransfected cells (UT) or cells expressing mCherry only, mCherry-tagged cyclin F^WT^ or mutant cyclin F^S621G^ for 48h. *Left and middle columns*: scale bar = 5 *µ*m. *Right column:* Higher magnification images acquired with Airyscan of the area delimited by white squares on images on middle column: scale bar = 0.5 µm^98^. As ERES generate COPII-coated vesicles with an average diameter of 60–90 nm^42^, each green dot represents clusters of Sec31-positive ERES, rather than a single Sec31-positive COPII vesicle. (**b**) Diameter of ERES clusters calculated from images obtained in (**a**). For mCherry, cyclin F^WT^ and cyclin F^S621G^ expressing cells, symbols represent individual experiments. ERES cluster diameter was calculated from n = 30 cells. Mean ± SEM. Symbols represent independent experiments; one-way ANOVA followed by a post-hoc Tukey test for multiple comparisons, *p < 0.05 vs untransfected cells and cells expressing either mCherry only or cyclinF^WT^. (**c**) Proportion of HEK293T cells with diameters of ERES clusters below 90 nm; between 90 and 120 nm; and over 120 nm. Mean ± SEM; symbols represent n = 3 independent experiments, **p < 0.01 *vs* untransfected cells (UT), mCherry only and cyclin F^WT^ cells, one-way ANOVA, followed by post-hoc Tukey test. The total number of ERES clusters quantified in each group across the three replicates was UT: n = 1827; mCherry: n = 2114; cyclin F^WT^: n = 1722; cyclin F^S621G^: n = 1746. (**d**) Size distribution curve of Sec31-positive ERES clusters in untransfected HEK293 cells and cells expressing mCherry or cyclin F. n = 3 independent experiments, 380 + ERES clusters per group.

COPII-coated vesicles form at ERES, where several hundred vesicles often cluster together^41^. Hence, ERES can be distinguished from the surrounding ER membranes by the presence of COPII proteins, including Sec31. We examined the morphology of Sec31-positive clusters in HEK 293 T cells using whole cell images and observed that most clusters (84.54 ± 0.03%) displayed larger diameters (> 118 nm) (Supplementary Fig. 3a-c). However, COPII vesicles are ~ 60–90 nm in diameter^42^, implying that individual vesicles and smaller clusters were not resolved by examining the whole cell. Hence, we examined the morphology of Sec31-positive clusters in HEK 293 T cells expressing mutant cyclin F by airyscan microscopy to examine these clusters with higher resolution (Fig. 3a–c). These clusters were identified as hollow, spherical structures staining positive for Sec31, with a diameter 60–350 nm. The mean diameter of ERES clusters was significantly decreased (1.2-fold, *p* < 0.05) in cells expressing cyclin F^S621G^, compared to untransfected cells or those expressing either mCherry or cyclin F^WT^ (Fig. 3b). Furthermore, significantly more ERES clusters with smaller diameters (90 nm) and conversely, significantly less ERES clusters with larger diameters (< 120 nm) were present in cells expressing cyclin F^S621G^ compared to untransfected cells, and mCherry or cyclin F^WT^ cells (Fig. 3c). This was reflected by a shift to the left when the size distribution of the diameters of Sec31-positive clusters was examined in cells expressing cyclin F^S621G^**,** compared to the other groups (Fig. 3d). These results suggest that COPII vesicular clusters at ERES are narrower and thus irregular in cells expressing variant cyclin F^621G^, consistent with inhibition of ER-Golgi trafficking.

### ALS/FTD-associated cyclin F^S621G^ induces ERAD

**Figure 4:**
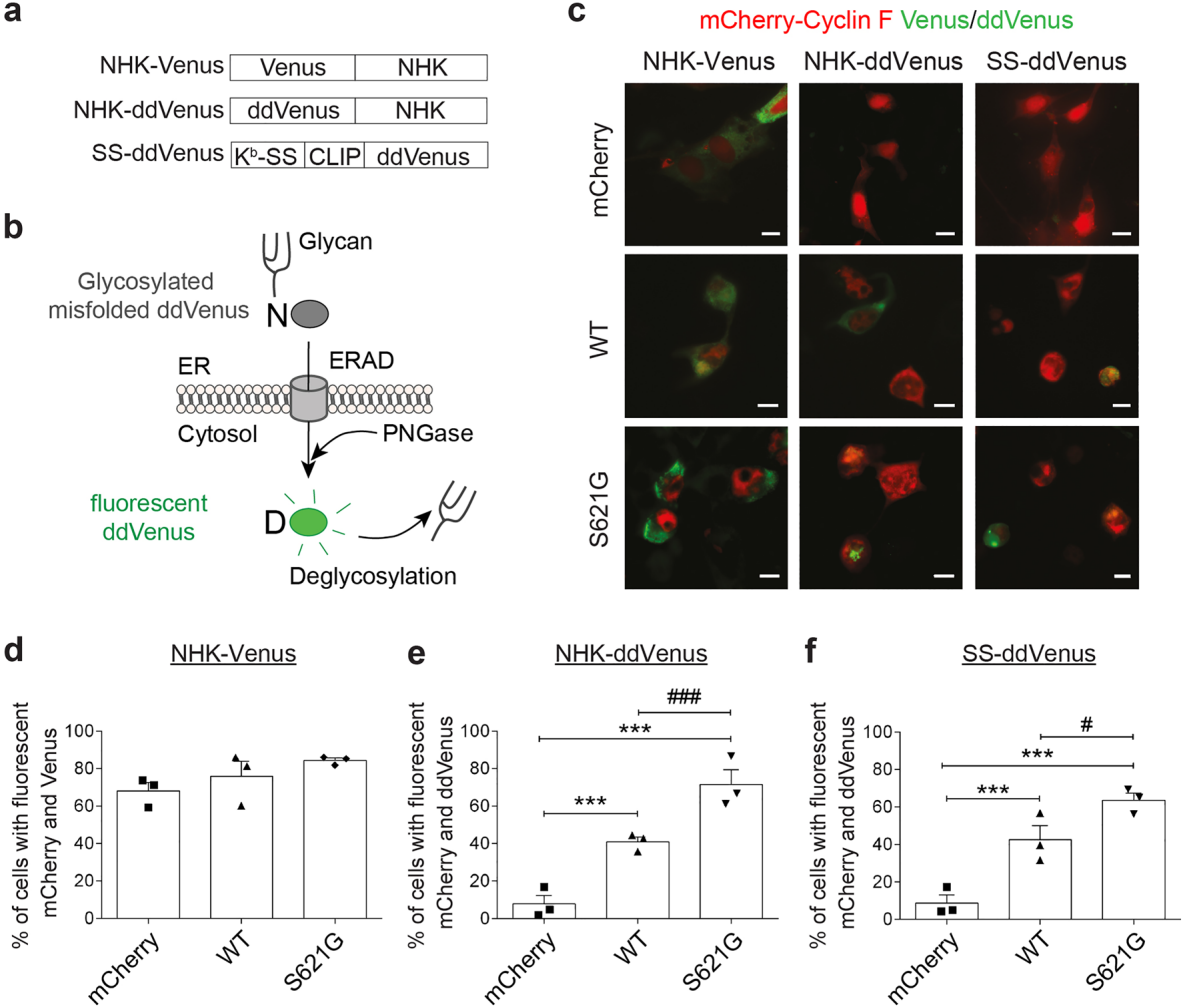
ALS/FTD variant cyclin F^S621G^ activates ERAD. (**a**) Fluorescent ERAD substrates used to monitor ERAD. Wild-type Venus and mutant ddVenus, in which Asn is substituted for Asp at position 82 (SS-ddVenus), were fused to a well-known ERAD substrate, the unstable null Hong Kong variant of alpha-1–antitrypsin (NHK-Venus; NHK-ddVenus)^25^. SS-ddVenus contains ER-targeted signal sequence (K^b^-SS) fused to dd-Venus. (**b**) The system is based on glycosylation and deglycosylation of Venus. Removal of a glycan by the cytosolic enzyme Peptide:N′glycanase (PNGase) results in deamidation of the glycosylated asparagine (N), converting it to an aspartic acid (D) residue, restoring fluorescence. The fluorescence results from protein that both reaches the ER and also retrotranslocates to the cytosol. Both glycosylation and deglycosylation (ER entry and exit) are required for fluorescence^25^. (**c**) Representative fluorescent microscopy merge images of SH-SY5Y cells co-expressing ERAD substrates (green) with either mCherry, cyclin F^WT^ or cyclin F^S621G^ (red). Arrows represent ddVenus fluorescent cells expressing cyclin F^S621G^. NHK fused to Venus was used as a control for cell transfection. Scale bar = 10 µm. (**d**–**f**) Graphs illustrate the proportion of cells co-expressing cyclin F with NHK-Venus (**d**), NHK-ddVenus (**e**) or SS-ddVenus (**f**) relative to the total number of mCherry-fluorescent cells. Graphs represent mean ± SEM. Symbols represent three independent experiments, ***p < 0.001 *vs* mCherry only; ^#^*p* < 0.05, ^###^*p* < 0.001 *vs* cyclin F^WT^, One-way ANOVA followed by post-hoc Tukey test for multiple comparisons.

Inhibition of the early stage of ER-Golgi transport by cyclin F^S621G^ implies that partially-folded proteins within the ER cannot efficiently exit this compartment, leading to their accumulation and hence ER stress. This would activate ERAD, an important quality control function to degrade misfolded ER proteins^43, 44^. Hence, we next examined ERAD in cyclin F cells. ERAD first involves the recognition of substrate, followed by ubiquitination, retro-translocation and then proteasomal degradation. We used a specific ERAD substrate with an ER-targeted signal sequence (K^b^-SS) fused to a Venus variant, ddVenus (deglycosylation-dependant Venus), where Asp is substituted to Asn at position 82^25^ (Fig. 4a). This mutation results in glycosylation and reduced fluorescence, which is restored to wildtype levels when Asn is converted back to Asp. Removal of oligosaccharides by endogenous peptide:N′glycanase (PNGase) in the cytosol results in deamidation of glycosylated Asn, converting it to Asp which restores fluorescence^25^ (Fig. 4b). As ERAD involves two stages—entry of substrate into the ER, followed by retro-translocation to the cytoplasm—both glycosylation of ddVenus in the ER and deglycosylation in the cytosol are required for fluorescence. Hence accumulation of ddVenus indicates ERAD activation. In addition, a second ERAD substrate was used, fluorescent ddVenus fused to the null Hong Kong genetic variant of α1-antitrypsin (NHK-ddVenus), which misfolds terminally in the ER and is also degraded specifically by ERAD^24, 25, 45^. In cells expressing mCherry only, cyclin F^WT^ or cyclin F^S621G^, ERAD was probed using substrates NHK-Venus (control for transfection efficiency), NHK-ddVenus or SS-ddVenus (Fig. 4c). Quantification demonstrated that significantly more fluorescent cells were present in cyclin F^S621G^ expressing populations compared to cyclin F^WT^ (NHK-ddVenus: 1.7-fold; SS-ddVenus: 1.5-fold,) or mCherry only (NHK-ddVenus: 33.1-fold; SS-ddVenus: 33.8-fold) (Fig. 4d–f). Hence, expression of cyclin F^S621G^ activates ERAD compared to cyclin F^WT^ and controls.

### ALS/FTD cyclin F^S621G^ induces ER stress in neuronal cells, primary neurons and iPSC-derived motor neurons

**Figure 5:**
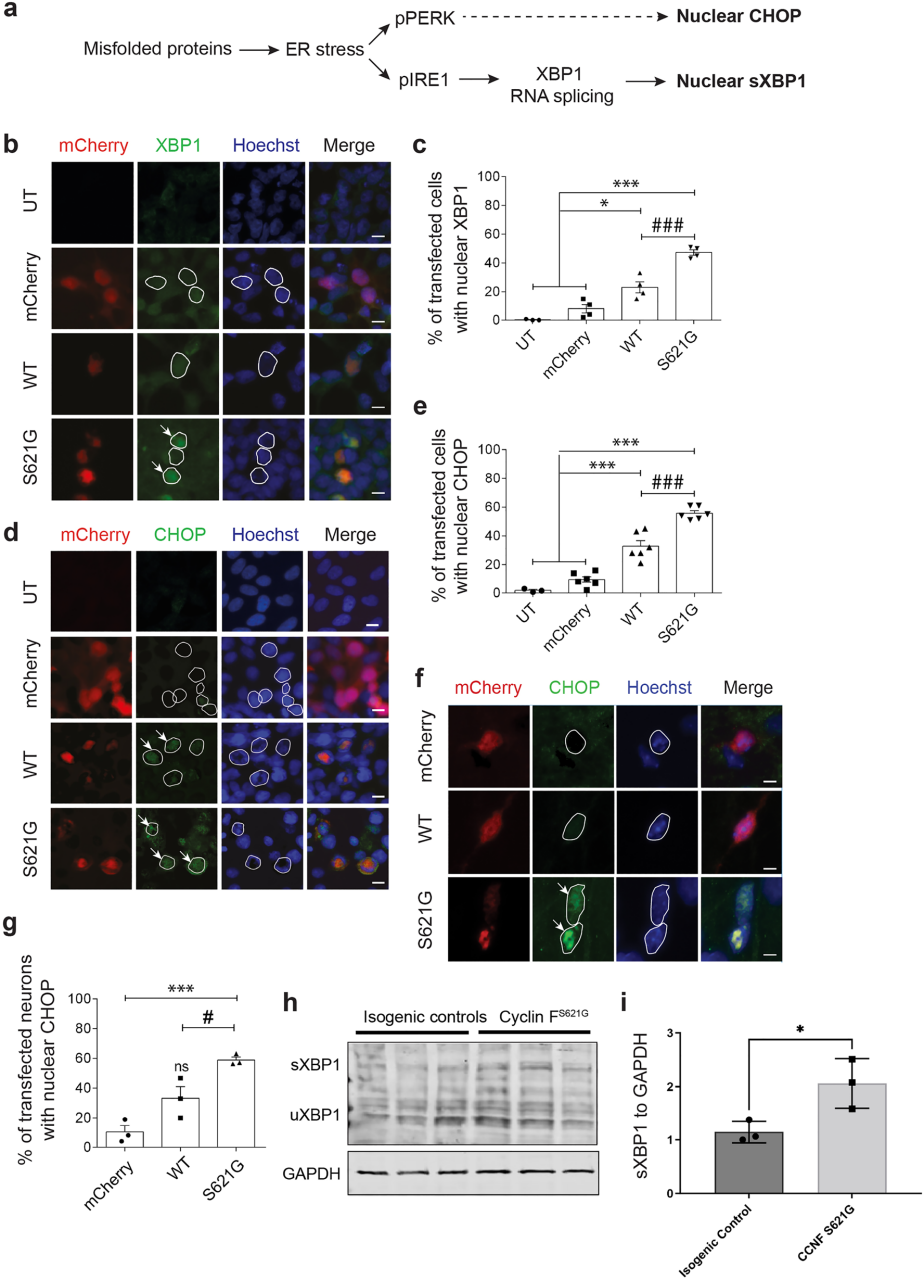
ALS/FTD-cyclin F^S621G^ induces ER stress in neuronal cells and cortical primary neurons (**c**, **e**, **g**) Symbols represent 3–6 independent experiments. (**a**) Schematic representing relationship between UPR markers. Misfolded proteins induce ER stress, activating PERK and IRE1α, leading to dimerization followed by phosphorylation. Phospho-IRE1 splices XBP1 (sXBP1) in the nucleus. Following chronic ER stress, CHOP is activated and translocates to the nucleus, inducing apoptosis. (**b**) Fluorescence microscopy following immunocytochemistry for XBP1 and Hoechst in HEK293T cells expressing mCherry, mCherry-tagged WT or cyclin F^S621G^. Nuclei: white outline. Arrows: nuclear XBP1 immunoreactivity. Scale bar = 10 μm. (**c**) Proportion of cells with nuclear XBP1 in (**b**). Cells undergoing apoptosis (with condensed nuclei) were excluded. Mean ± SEM, n = 100 + cells/group. One-way ANOVA, post-hoc Tukey test; *p < 0.05, ***p < 0.001 *vs* untransfected (UT) and mCherry only cells; ^###^*p* < 0.001 *vs* cyclin F^WT^cells. (**d**) Fluorescence microscopy following immunocytochemistry for CHOP and Hoechst in HEK293T cells expressing mCherry, mCherry-tagged WT or cyclin F^S621G^. Nuclei: white outline. Arrows: nuclear CHOP immunoreactivity. Scale bar = 10 μm. (**e**) Proportion of cells with nuclear CHOP in (**d**). Cells undergoing apoptosis (with condensed nuclei) were excluded. Mean ± SEM; n = 100 + cells/group. One-way ANOVA, post-hoc Tukey test; ***p < 0.001 *vs* untransfected (UT) and cells expressing mCherry; ^###^*p* < 0.001 *vs* cells expressing cyclin F^WT^. (**f**) Fluorescence microscopy of mouse primary cortical neurons expressing mCherry only, or mCherry-tagged cyclin F, following immunocytochemistry for CHOP and Hoechst. Nuclei: white outline. Arrows: nuclear CHOP immunoreactivity. Scale bar = 10 *μ*m. (**g**) Proportion of neurons with nuclear CHOP in (**f**). Neurons undergoing apoptosis (with condensed nuclei) were excluded. Mean ± SEM; n = 10–30 neurons/group; one-way ANOVA, post-hoc Tukey test. ns, non-significant (WT vs mCherry), ***p < 0.001 *vs* mCherry only; ^#^*p* < 0.05 *vs* cells expressing cyclin F^WT^. (**h**) Western blotting of sXBP1 in lysates from iPSC-derived motor neurons from a cyclin F^S621G^ patient and CRISPR-corrected isogenic control, GAPDH as loading control. uXBP1, unspliced XBP1 (**i**) sXBP1 expression from (**h**) relative to GAPDH. Three iPSC differentiation replicates for both cyclin F^S621G^and isogenic control were analysed on the same blot, with two technical replicate blots. The mean sXBP1 intensity in cyclin F^S621G^ relative to GAPDH per blot is shown, all samples were normalised to the first differentiation isogenic control replicate. Mean ± SD; paired t-test, *p < 0.05.

These results imply that cyclin F^S621G^ induces ER stress. To investigate this possibility further, we examined whether cyclin F^S621G^ activates UPR following established methods^46–48^ (Fig. 5). During ER stress, misfolded proteins accumulate in the ER, which activates and phosphorylates UPR sensors, including PERK and IRE1. The latter activates transcription factor XBP1, which becomes spliced in the nucleus (forming spliced XBP1, sXBP1) (Fig. 5a). Following chronic ER stress, transcription factor CHOP is activated only when the UPR becomes pro-apoptotic^49^. Both XBP-1 and CHOP translocate to the nucleus when activated during ER stress. Hence, splicing of XBP-1 and nuclear immunoreactivity to both XBP-1 and CHOP therefore indicates activation of pro-apoptotic phases of the UPR (Fig. 5a). Expression of cyclin F^S621G^ significantly increased the proportion of cells with nuclear XBP1 immunoreactivity, compared to cells expressing cyclin F^WT^ (twofold), mCherry only (5.9-fold) or untransfected cells (173-fold) (Fig. 5b,c), implying that cyclin F^S621G^ induces ER stress. This was confirmed following immunocytochemistry for CHOP. Significantly more cells with nuclear CHOP immunoreactivity were detected in populations expressing variant cyclin F^621G^ compared to cells expressing cyclin F^WT^ (1.7-fold), mCherry only (5.9-fold) or untransfected cells (31.4-fold; Fig. 5d,e). The proportion of untransfected cells and mCherry-expressing cells with XBP1 or CHOP immunoreactivity was not significantly different (Fig. 5d,e), confirming that ER stress was not induced by non-specific protein overexpression. Compared to mCherry, expression of cyclin F^WT^ resulted in significantly more cells with nuclear immunoreactivity to CHOP, but not to XBP1, implying that wildtype cyclin F also induces low levels of ER stress, albeit significantly less than the mutant. These results indicate that ALS/FTD-associated cyclin F^S621G^ induces ER stress in neuronal cell lines.

This finding raises the question of whether ER stress is induced directly, by the presence of cyclin F^S621G^ in the ER, or indirectly, by cyclin F^S621G^ localised in the cytoplasm. Dysregulation of ER-Golgi trafficking can induce ER stress by cytoplasm-located proteins^50^, including those associated with ALS^14^. Hence, we next examined whether cyclin F^S621G^ is present in the ER, which would imply it directly triggers ER stress. Subcellular fractionation of lysates to produce ER-rich fractions was performed. Western blotting for calnexin confirmed the presence of ER membranes (Supplementary Fig. 4a) and blotting for cyclin F revealed similar levels of both cyclin F^WT^ and cyclin F^S621G^ in the ER fractions (Supplementary Fig. 4b). To further confirm these findings, immunocytochemistry for calnexin was performed. Analysis using Mander’s coefficient (where 0 = no co-localisation and 1 = high co-localisation) demonstrated little co-localisation between wildtype (0.3) or cyclin F^S621G^ and calnexin (0.2). Furthermore, no significant differences were detected between localisation of calnexin with either cyclin F^WT^ or cyclin F^S621G^ (Supplementary Fig. 4c,d), despite differences in induction of ER stress by these proteins. Hence, these results imply that ALS/FTD cyclin F^S621G^ does not induce ER stress by excess accumulation of cyclin F in the ER.

To confirm the findings obtained in cell lines, ER stress was next examined in mouse primary cortical neurons expressing cyclin F^S621G^ by immunocytochemistry (Fig. 5f). Quantification demonstrated that significantly more primary neurons expressing cyclin F^S621G^ displayed nuclear immunoreactivity to CHOP (1.7-fold), compared to cyclin F^WT^ or mCherry only cells (Fig. 5f,g). Similarly, nuclear localisation of XBP-1 was detected in primary neurons expressing cyclin F^S621G^ unlike controls (Supplementary Fig. S5), although quantification could not be performed due to low transfection efficiency. Hence, cyclin F^S621G^ activates ER stress in primary cortical neurons, confirming the results obtained in cell lines.

Finally, we also examined iPSC-derived motor neurons from an ALS patient carrying the cyclin F^S621G^ mutation^27^ and the equivalent CRISPR-corrected isogenic control cells (cyclin F wild-type), after 12 days of maturation. Cell lysates from these cells were subjected to western blotting for XBP1, revealing that expression of spliced XBP-1 was significantly increased in iPSC-derived motor neurons expressing cyclin F^S621G^ compared to the isogenic control (Fig. 5h,i, 1.8-fold). Hence these findings provide further evidence that cyclin F^S621G^ activates ER stress.

### ALS/FTD cyclin F^S621G^ induces fragmentation of the Golgi

**Figure 6:**
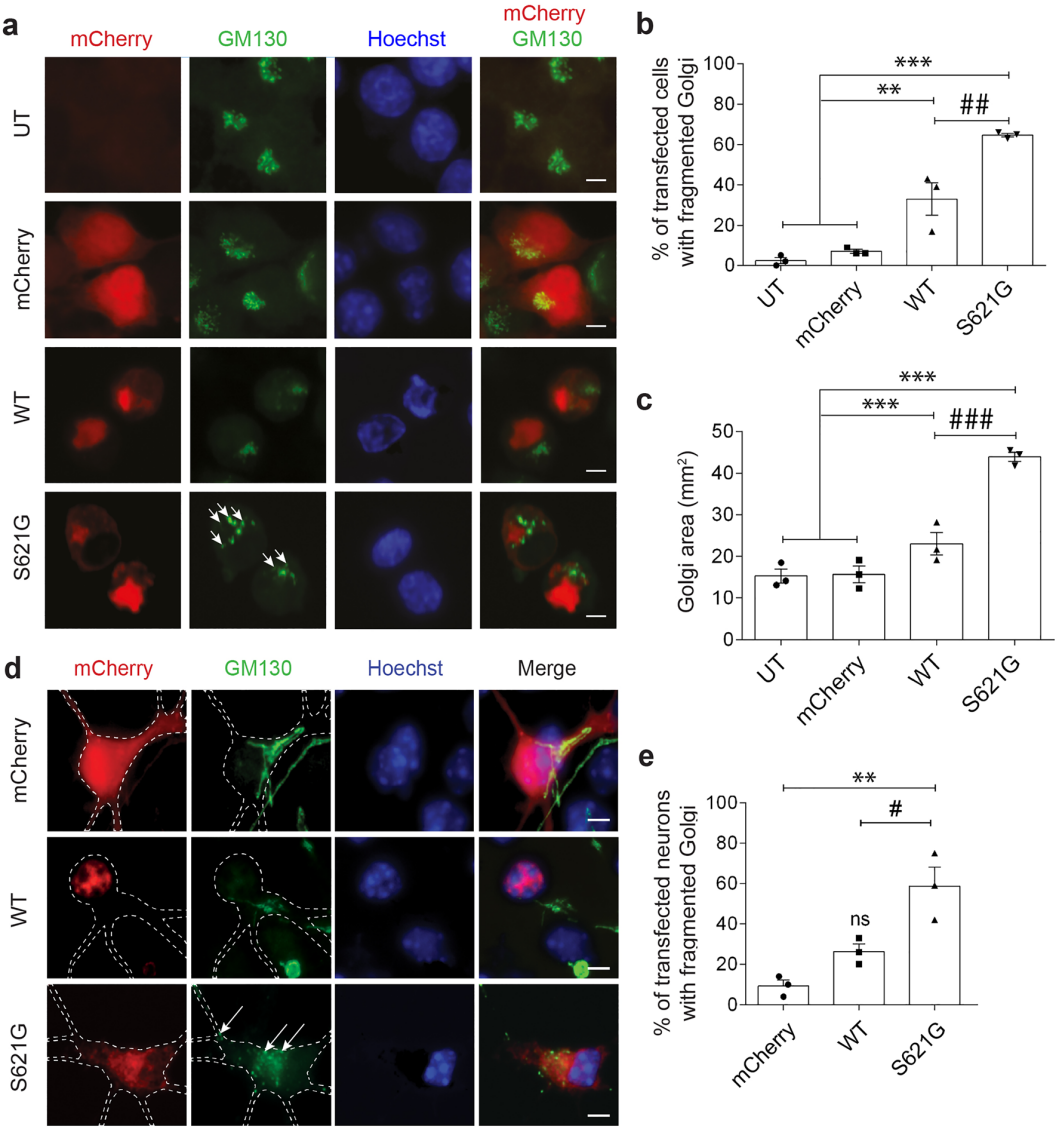
ALS/FTD cyclin F^S621G^ induces Golgi fragmentation. (**a**) Fluorescent confocal microscopy images following immunocytochemistry for GM130 and Hoechst staining, of SH-SY5Y cells expressing mCherry only or mCherry-tagged cyclin F WT or variant S621G. Arrows: fragmented Golgi. Apoptotic cells displaying a condensed nucleus were excluded from the analysis. Scale bar = 10 µm. (**b**) The proportion of cells with fragmented Golgi in (**a**) was quantified. Apoptotic cells displaying a condensed nucleus were excluded from analysis. Mean ± SEM. Symbols represent 3 independent experiments, n = 50 + cells per group from, one-way ANOVA, post-hoc Tukey test for multiple comparisons; **p < 0.01 and ***p < 0.001 *vs* untransfected cells (UT) and mCherry only; ^##^*p* < 0.01 *vs* cells expressing cyclin F^WT^. (**c**) The area covered by fragmented Golgi was quantified per cell using the images in (**a**). Mean ± SEM. Symbols represent 3 independent experiments, n = 50 + cells per group one-way ANOVA followed by a post-hoc Tukey test for multiple comparisons; ns, non-significant (WT vs UT and mCherry), ***p < 0.001 *vs* UT and mCherry; ^###^*p* < 0.01 *vs* cells expressing cyclin F^WT^. (**d**) Fluorescent confocal microscopy images of following immunocytochemistry for GM130 and Hoechst staining in mouse primary cortical neurons expressing mCherry only or cyclin F (WT or S621G). Arrows: fragmented Golgi. The dashed white line delimits the outline of the neuron. Scale bar = 5 µm. (**e**) The proportion of primary neurons with fragmented Golgi was quantified. Apoptotic neurons displaying a condensed nucleus were excluded from the analysis and thus are not shown here. Mean ± SEM. Symbols represent 3 independent experiments, n = 10–30 neurons per group; one-way ANOVA followed by a post-hoc Tukey test for multiple comparisons; ns, non-significant (WT vs UT), **p < 0.01 *vs* mCherry; ^#^*p* < 0.05 *vs* cells expressing cyclin F^WT^.

Golgi fragmentation follows ER stress^51, 52^, and inhibition of ER-Golgi trafficking^14, 53–55^, raising the possibility that cyclin F^S621G^ also induces this phenotype. Hence, next the morphology of the Golgi apparatus was examined by immunocytochemistry for GM130 as a marker. In SH-SY5Y (Fig. 6a) and HEK293T (Supplementary Fig. 6a, b) cells expressing mCherry only or cyclin F^WT^, the Golgi displayed its typical morphology of continuous, stacked membranous cisternae. However, in cyclin F^S621G^ expressing HEK293T cells and SH-SY5Y cells, the Golgi apparatus was fragmented, displaying multiple disconnected elements or tubular-vesicular clusters^56^. Quantification revealed that significantly more cells with fragmented Golgi were present in populations expressing cyclin F^S621G^ (HEK293T: 1.5-fold; SH-SY5Y: twofold) compared to cyclin F^WT^, mCherry or untransfected cells (Fig. 6b, Supplementary Fig. 6).

To provide evidence that Golgi fragmentation correlates with impairment of ER-Golgi transport, we next examined Golgi morphology in the GM130-immunostained SH-SY5Y cells used in the VSVG^ts045^ assay (Fig. 1). Quantification revealed that significantly more cyclin F^S621G^ cells displayed fragmented Golgi compared to cyclin F^WT^ (2.6-fold) or mCherry- cells (13.2-fold change, Supplementary Fig. 7a). The proportion of cells co-expressing VSVG^ts045^ and cyclin F with Golgi fragmentation (57.3 ± 8.6%) was also similar to results obtained in Fig. 6b (64.6 ± 1%). Hence Golgi fragmentation is present in cells in which VSVG^ts045^ secretion is impaired, confirming that inhibition of ER-Golgi transport correlates with fragmentation of the Golgi.

To further confirm these results, a more unbiased quantification method was used. Golgi stacks can be dispersed (mini-stacks) or completely disassembled, hence the surface area covered by the Golgi also indicates fragmentation^51^. Quantification revealed a significant increase in Golgi fragment surface area in cells expressing cyclin F^S621G^ (1.9-fold) compared to cyclin F^WT^ and control untransfected or mCherry cells (Fig. 6c). Similarly, we also examined the GM130-immunostained cells prepared for the VSVG^ts045^ assay (Fig. 1). Quantification revealed that a significant increase in the area covered by fragmented Golgi in cells expressing cyclin F^S621G^ compared to cyclin F^WT^ (2.6-fold) or mCherry cells (13.5-fold, Supplementary Fig. 7b) (57.4%), similar to Fig. 6 (64.7%). Together these results demonstrate that cyclin F^S621G^ induces Golgi fragmentation in neuronal cells, which correlates with inhibition of ER-Golgi transport.

To further confirm the above results, we next examined mouse cortical primary neurons expressing cyclin F. Immunocytochemistry was performed for GM130 to examine the morphology of the Golgi as above (Fig. 6d). Significantly more neurons expressing cyclin F^S621G^ displayed Golgi fragmentation compared to those expressing WT (2.2-fold) and mCherry (6.3-fold) (Fig. 6e). Hence, ALS/FTD-associated cyclin F^S621G^ induces Golgi fragmentation in primary cortical neurons, confirming the results obtained in cell lines.

### ALS/FTD mutant cyclin F induces cell death

**Figure 7:**
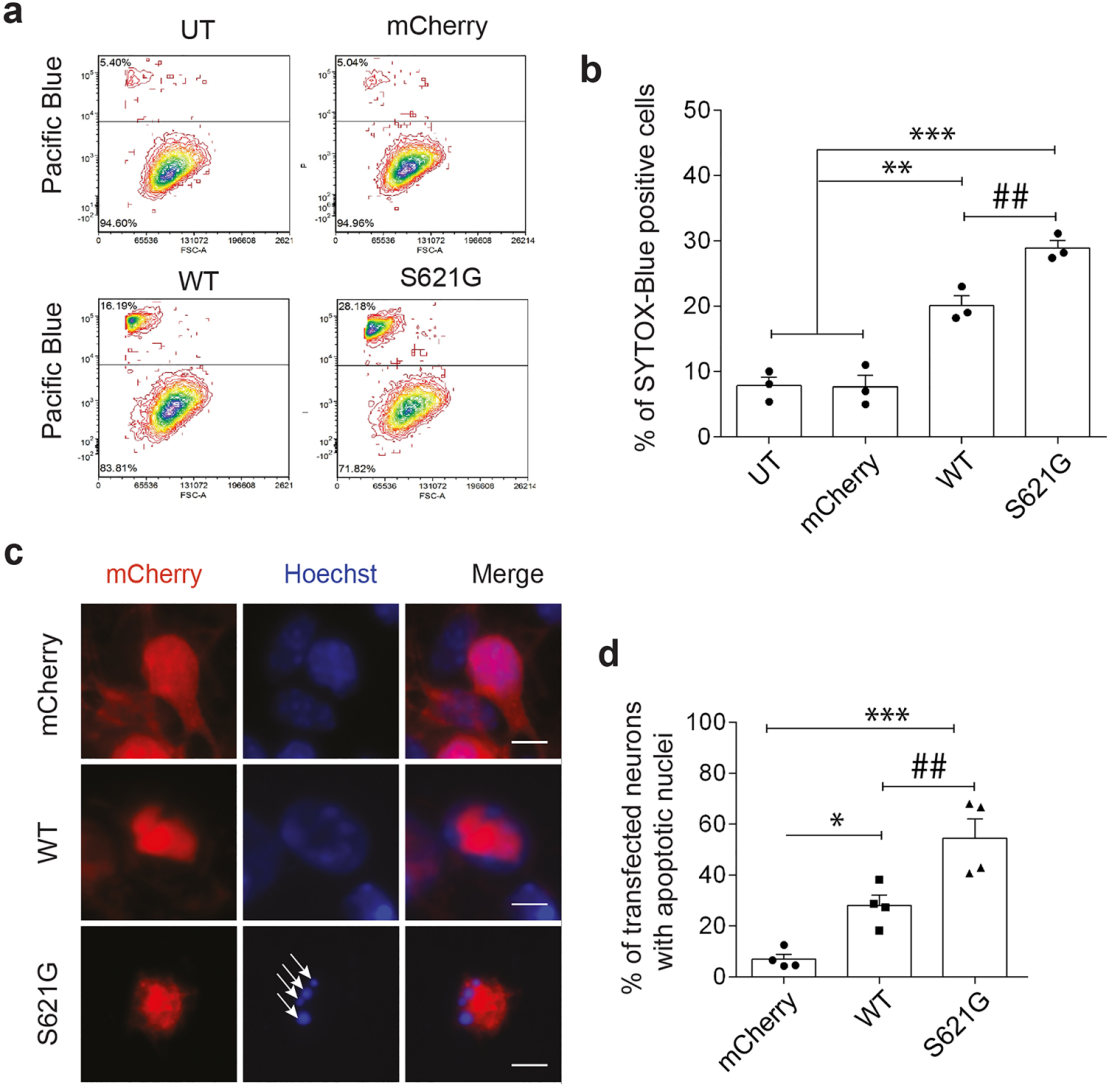
ALS/FTD cyclin F^S621G^ induces cell death. (**a**) Flow cytometry analysis of SH-SY5Y cells expressing mCherry-tagged cyclin F after staining with Sytox Blue, a marker for dead cells*.* UT, untransfected cells, mCherry only, cyclinF^WT^, variant cyclinF^S621G^. (**b**) Quantification of Sytox Blue positive cells in (**a**). Mean ± SEM. Symbols represent n = 3–4 independent experiments, one-way ANOVA, followed by a post-hoc Tukey test for multiple comparisons, **p < 0.001, ***p < 0.001 compared to untransfected cells (UT) and mCherry only; ^##^*p* < 0.05 compared to cyclin F^WT^. (**c**) Representative fluorescent microscopy images of mouse cortical primary neurons expressing cyclin F, stained with Hoechst. Condensed nuclei (arrows), indicative of neuronal death, are present in cells expressing variant cyclin F. Scale bar = 10 µm. (**d**) The proportion of cells with condensed, fragmented nuclei in (**c**) was quantified. Mean ± SEM. Symbols represent n = 3–4 independent experiments, one-way ANOVA, followed by post-hoc Tukey test for multiple comparisons, *p < 0.05, ***p < 0.001 compared to mCherry only, ^##^*p* < 0.01 compared to cyclin F^WT^.

ER stress induces apoptosis when prolonged/severe, and Golgi fragmentation also triggers apoptosis^57, 58^. Hence cell death was next analysed by flow cytometry following Sytox Blue staining, in SH-SY5Y cells expressing cyclin F (Fig. 7a,b). Quantitative analysis demonstrated significantly more dead cells in populations expressing cyclin F^S621G^ compared to untransfected cells (3.7-fold) and those expressing cyclin F^WT^ (1.4-fold) or mCherry (3.8-fold), indicating that cyclin F^S621G^ expression induces cell death.

This was next examined in primary neurons, where the presence of condensed nuclear morphology indicated induction of apoptosis, as previous^46, 59^. Quantitative analysis of primary neurons expressing cyclin F demonstrated that significantly more neurons were undergoing apoptosis in populations expressing cyclin F^S621G^ compared to wildtype (1.9-fold) or mCherry (cyclin F^S621G^: 7.9-fold) (Fig. 7c,d). Hence, these data confirm that ALS/FTD-cyclin F^S621G^ induces apoptosis.

Finally, we also examined a second ALS/FTD-associated variant, p.S195R, to probe whether ER/Golgi perturbations are specific to cyclin F^S621G^ only. Golgi morphology was examined as a marker of trafficking dysfunction in SHSY5Y cells using GM130. Significantly more Golgi fragmentation (1.6-fold) was detected when cyclin F^S195R^ was expressed, compared to cyclin F^WT^, mCherry and untransfected cells (Supplementary Fig. 8a–c). Similarly, significantly more primary neurons expressing cyclin F^S195R^ displayed Golgi fragmentation compared to those expressing WT (2.9-fold) and mCherry (8.1-fold) (Supplementary Fig. 8d–e). Hence the Golgi is fragmented by ALS-FTD-associated mutant F^S195R^. Similarly, significantly more primary neurons expressing cyclin F^S195R^ displayed nuclear immunoreactivity to CHOP (2.2-fold), compared to cyclin F^WT^ or mCherry only expressing cells (Supplementary Fig. 8 f–g), indicating activation of ER stress. Consistent with this result, significantly more neurons bearing apoptotic nuclei were present in cyclin F^S195R^ populations compared to cyclin F^WT^ (1.8-fold) or mCherry (7.3-fold) (Supplementary Fig. 8h–i). There were also similar levels of cyclin F^S195R^ in the ER-rich fractions compared to both cyclin F^WT^ or cyclin F^S621G^ (Supplementary Fig. 4a,b). Hence these findings imply that the second ALS/FTD variant also perturbs ER-Golgi homeostasis.

## Discussion

This study demonstrates that ALS/FTD-associated cyclin F^S621G^ perturbs ER homeostasis by inhibiting an important ER function: transport of secretory/transmembrane proteins to the Golgi. This induces Golgi fragmentation, ER stress, ERAD, and apoptosis, in both cell lines and mouse cortical primary neurons. Together these data imply that ER dysfunction is important in neurodegeneration induced by cyclin F^S621G^, thus providing novel insights into *CCNF*-associated ALS/FTD.

ER-Golgi transport is a vital gateway to the endomembrane system, and one-third of all proteins transit via this pathway before reaching their final locations^18, 60^. Our results imply that ALS/FTD-associated cyclin F^S621G^ perturbs the first stage of ER-Golgi transport: budding of COPII vesicles from the ER. The COPII coat is essential for the formation of transport vesicles on the cytosolic face of the ER membrane. Curvature of the ER membrane, concentration of cargo and vesicular release then results. Defective COPII vesicles are known to inhibit secretion^20^. Here we detected several abnormalities in COPII in cyclin F^S621G^ cells, less vesicular cargo (VSVG^ts045^) and narrower Sec31-positive clusters, demonstrating that the ERES are abnormal. Typical COPII vesicles are 60–90 nm in diameter, and it was not possible to resolve individual COPII vesicles or ERES using the methods used here. The vesicle clusters were found to contain smaller diameters in mutant cyclin F expressing cells, implying that they are irregular and possibly misshapen. Furthermore, whilst Sec23 levels correlate with the load of protein cargo^20^, here the levels of Sec23 were comparable to cargo load, suggesting that the defect is related to COPII vesicles themselves, rather than the mechanisms involving incorporation of protein cargo. Hence, these results imply that there is a defect in the formation of COPII vesicles and/or ERES in cyclin F^S621G^ expressing cells.

Ubiquitination of Sec31 (by the CUL3-KLHL12 complex) facilitates the production of large COPII vesicles to transport bulky cargo^20, 61^ by a calcium-dependant mechanism^20, 62^. Similarly, deubiquitylation of Sec31 antagonizes the formation of large pro-collagen-containing carriers^63^. We previously demonstrated that cyclin F^S621G^ dysregulates ubiquitination at Lys48, disrupting cellular survival and maintenance networks^9, 11^. Hence it is possible that aberrant ubiquitination of Sec31 in cyclin F^S621G^ cells impairs the formation of COPII vesicles, forming narrower ERES clusters, as detected here. However, future studies are required to examine this possibility. Collagen is the most abundant cargo for COPII vesicles^63^, and whilst it was previously thought to be absent in the brain^64^, neurons are now known to express collagen^65, 66^. Whilst poorly understood, collagen displays functions in both the PNS and CNS^67^. It regulates axonal outgrowth and synaptic differentiation^68^, and is dysregulated during stress and in Alzheimer’s disease^67^. Mutations in the genes encoding Sec23A^69^ or Sar1B^70^ lead to accumulation of large proteins (including collagen) and lipid particles in the ER^69^ in Skull-Lenticular-Sutural Dysplasia^69^ and Chylomicron Retention Disease^70^ respectively. This highlights the importance of COPII vesicle integrity in regulation of protein secretion, which when dysregulated, leads to pathological outcomes.

Impairment of ER-Golgi and other forms of transport has been previously described in ALS^14, 71–74^, in cells expressing SOD1^14^, TDP-43^14^, FUS^14^ or ubiquilin2 variants^15^. Moreover, this was previously linked to ER stress^14^, which is widely implicated in ALS in association with *SOD1*^46^, *TARDBP*^75^, *FUS*^76^, *OPTN*^77–79^, *VCP*^80^, *UBQLN2*^15^, *VAPB*^81, 82^ and *C9orf72*^83^. Here we detected UPR activation by three separate markers, spliced XBP1, IRE1 and CHOP. We also detected ER stress in iPSC-derived motor neurons from an ALS patient bearing a cyclin F^S621G^ mutation. In addition, we detected activation of ERAD, which is triggered during UPR activation, and which is also regulated by ubiquitination^84, 85^. Consistent with activation of pro-apoptotic CHOP during ER stress, expression of ALS/FTD cyclin F^S621G^ induced neuronal death. This finding is consistent with apoptotic death observed in the transient fish models overexpressing cyclin F^S621G^ and activation of caspase-3 in Neuro2A cells expressing cyclin F^S621G^^11^.

Previously ALS-variants of SOD1 or TDP-43 were shown to induce ER stress from the cytoplasm^71, 72, 86^, or from the cytoplasmic face of the ER^14^ respectively, by inhibiting ER-Golgi transport^14^. Here we detected similar expression of cyclin F^S621G^ and cyclin^WT^ in the ER by both immunocytochemistry and subcellular fractionation, raising the question of how ER stress is induced in ALS/FTD. As ALS-cyclin F mislocalizes to the cytoplasm, where it promotes the cytoplasmic aggregation of TDP-43^87^, it is possible that cyclin F^S621G^ induces ER stress from the cytoplasm by inhibiting ER-Golgi transport. However, we cannot rule out the possibility that cyclin F^S621G^ directly induces ER stress from the ER itself. Given that the cytosol and ER are closely associated, it is hard to conclusively determine whether a fraction of a cytosolic protein is bound to the ER membrane using immunocytochemistry. Furthermore, the ER-rich fractions isolated displayed weak reactivity to GAPDH (Supplementary Fig. 3a), indicating possible low levels of contamination from the cytoplasm. Hence further studies are required to confirm the contribution of both cytosolic and ER-localised to induction of ER stress by mutant cyclin F.

Golgi fragmentation is also well-described in ALS^23, 56, 88^, resulting in morphological changes and disruption of its characteristic ribbon-like structure^88, 89^. Fragmentation of the Golgi apparatus is present in sporadic ALS patients^90^ and before disease onset in SOD1^G93A^ mice^91^, prior to neuromuscular denervation and axon retraction^92^. It is also a feature of other neurodegenerative diseases^88^, but it has not been previously described for cyclin F in ALS. Here, we demonstrate that ALS/FTD-associated cyclin F variants trigger Golgi fragmentation. Proper organization of the Golgi depends on efficient bidirectional ER-Golgi vesicle transport^89, 93^ and both membrane flow and cargo load influence its structure and function. Hence, blocking export of cargo-containing ER carriers^94, 95^ or depleting cargo receptors^96^ results in Golgi fragmentation. Also the tubulo-vesicular Golgi clusters can further fuse with the ER, increasing ER stress^97^. In addition, inhibition of intra-Golgi trafficking or vesicle transport from the Golgi to the plasma membrane induces Golgi fragmentation if prolonged^53–55^. Hence, in this study, it is possible that the inhibition of ER-Golgi transport by cyclin F^S621G^ triggers Golgi fragmentation and ER stress. However, other stressors can induce Golgi fragmentation, so the directionality of these links cannot be conclusively established.

We also examined a second ALS/FTD variant in this study. In neuronal cells or primary neurons expressing cyclin F^S195R^, Golgi fragmentation and ER stress, leading to apoptosis, was detected. This implies that the trafficking perturbations induced by cyclin F^S621G^ are shared by other variants in ALS. However further studies are required to confirm this possibility.

**Figure 8:**
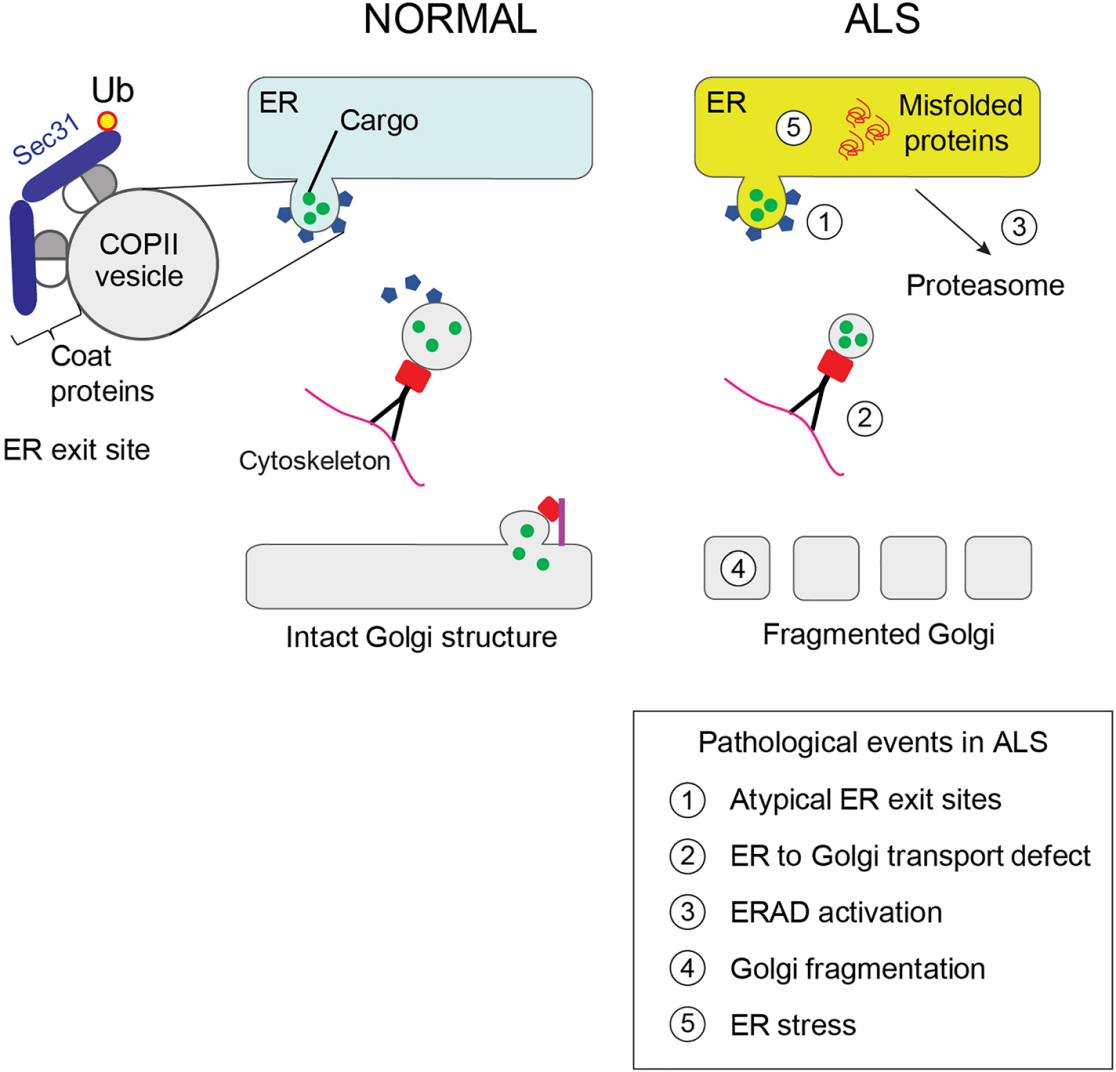
Schematic diagram based on this study, illustrating a possible mechanism to explain how mutant cyclin F impairs ER homeostasis in neuronal cells. Misfolded cyclin F triggers the formation of abnormal ER-derived COPII vesicles at ERES (1), which inhibits ER-Golgi transport (2), triggering ERAD (3), Golgi fragmentation (4), and ER stress (5), eventually resulting in apoptotic cell death.

In summary, this study identifies novel cellular mechanisms triggered by ALS/FTD-associated variant cyclin F^S621G^. In Fig. 8, we provide one hypothetical model to illustrate how these events are triggered. However, it is also possible that ER stress or Golgi fragmentation is the upstream trigger, which would subsequently impair ER-Golgi transport, given these events are all closely related. Alternatively, it is possible that the cellular events detected in this study result from a combination of these defects. Further studies are therefore required to probe the directionality of these links.

### Supplementary Information

Supplementary Figures.

## Data Availability

The datasets generated during and analysed during the current study are shown in the manuscript.

## Abbreviations

ALS: Amyotrophic lateral sclerosis
BSA: Bovine serum albumin
CUL1: Cullin-1
COPII: Coat protein complex II
ER: Endoplasmic reticulum
ERAD: Endoplasmic reticulum associated degradation
ERES: ER exit sites
FALS: Familial ALS
FTD: Frontotemporal dementia
NLS: Nuclear localization signals
PFA: Paraformaldehyde
SALS: Sporadic ALS
SKP1: S-phase kinase-associated protein 1
SEM: Standard error of the mean
UPR: Unfolded protein response
UPS: Ubiquitin proteasome system
UT: Untransfected
VSVG: Vesicular stomatitis viral glycoprotein
WT: Wild-type
