# Supplementary material for: ALS/FTD-associated mutation in cyclin F inhibits ER-Golgi trafficking, inducing ER stress, ERAD and Golgi fragmentation
Source: Sci Rep. 2023 Nov 22;13:20467. doi: 10.1038/s41598-023-46802-9 (PMC10665471; doi:10.1038/s41598-023-46802-9)

# Supplementary Information

Supplementary Figure 1 **Control experiments illustrating that VSVG^ts045^ is retained in the ER at 40°C.**

(**a)** Experimental paradigm for control cells: cells expressing VSVG^ts045^ for 24h were incubated at 40°C overnight (ON) to misfold VSVG^ts045^, leading to its accumulation in the ER. Cells were incubated for 30 min in cycloheximide (CHX) to inhibit further VSVG^ts045^ protein synthesis, but then maintained at 40°C instead of switching to the permissive temperature (32°C).

(**b**) Fluorescent microscopy images following immunocytochemistry for calnexin (an ER marker) in SH-SY5Y cells co-expressing GFP-tagged VSVG^ts045^ and either mCherry only (mCherry), cyclin F ^WT-^mCherry (WT) or variant cyclin F^S621G^ mCherry (S621G), following the experimental paradigm in (**a**). Scale bar = 5 *µ*m.

(**c**) The degree of co-localisation of VSVG^ts045^ with calnexin from the images in (**b**) was quantified using Mander’s coefficient, Mean ± SEM, one-way ANOVA followed by a post-hoc Tukey test for multiple comparisons, ns, non-significant.


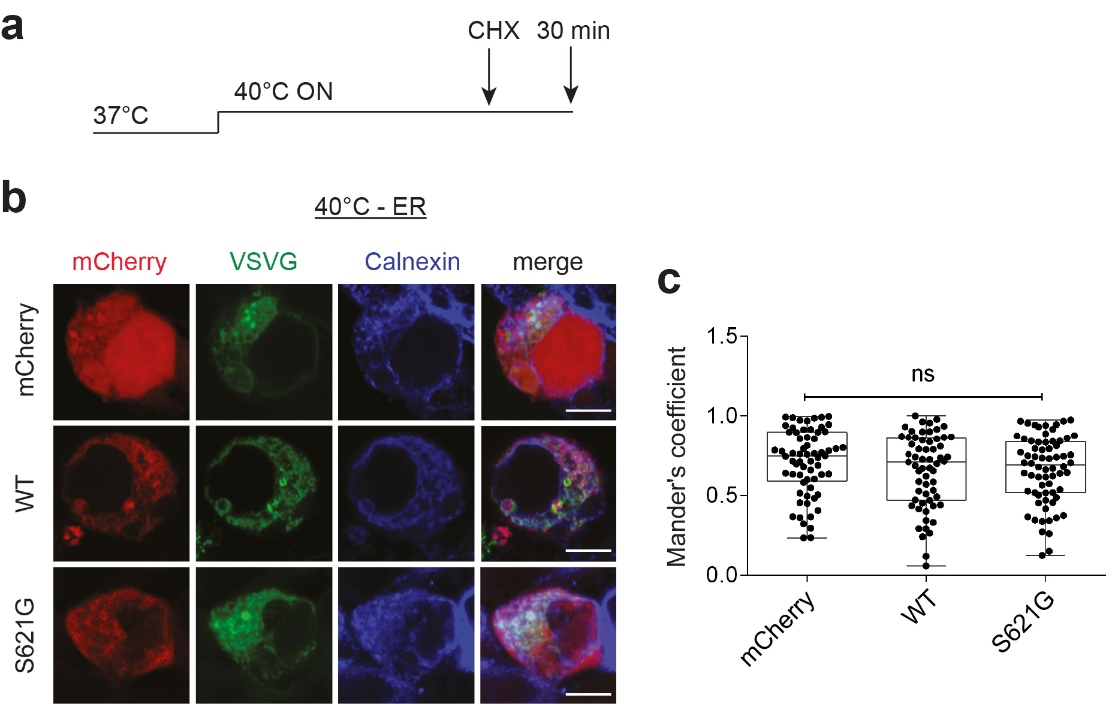


Supplementary Figure 2 **Control experiments for COPII vesicle budding assay.**

(**a**) Western blotting for VSVG with β-actin as a loading control of cells incubated at 40^o^C instead of the permissive temperature.

(**b**) Western blotting for Sec 23 with β-actin as a loading control of cell lysates sampled before the budding reaction. ns, non-significant.

(**c**) Quantification of blots in (**b**), normalised to β-actin as a loading control and untransfected cells (UT).

(**d**) Western blotting for Sec 23 with β-actin as a loading control of ER-derived budded vesicular fractions, sampled after the budding reaction. This is the same membrane as that

shown in Figure 2e (see uncropped blots), which was probed for both VSVG and

Sec23. Hence the same β-actin blot is shown for each.

(**e**) Quantification of blots in (**d**), normalised to β-actin as a loading control and untransfected cells (UT). Mean ± SEM. Symbols represent independent experiments, one-way ANOVA followed by a post-hoc Tukey test for multiple comparisons, **p*<0.05 *vs* untransfected cells and mCherry.


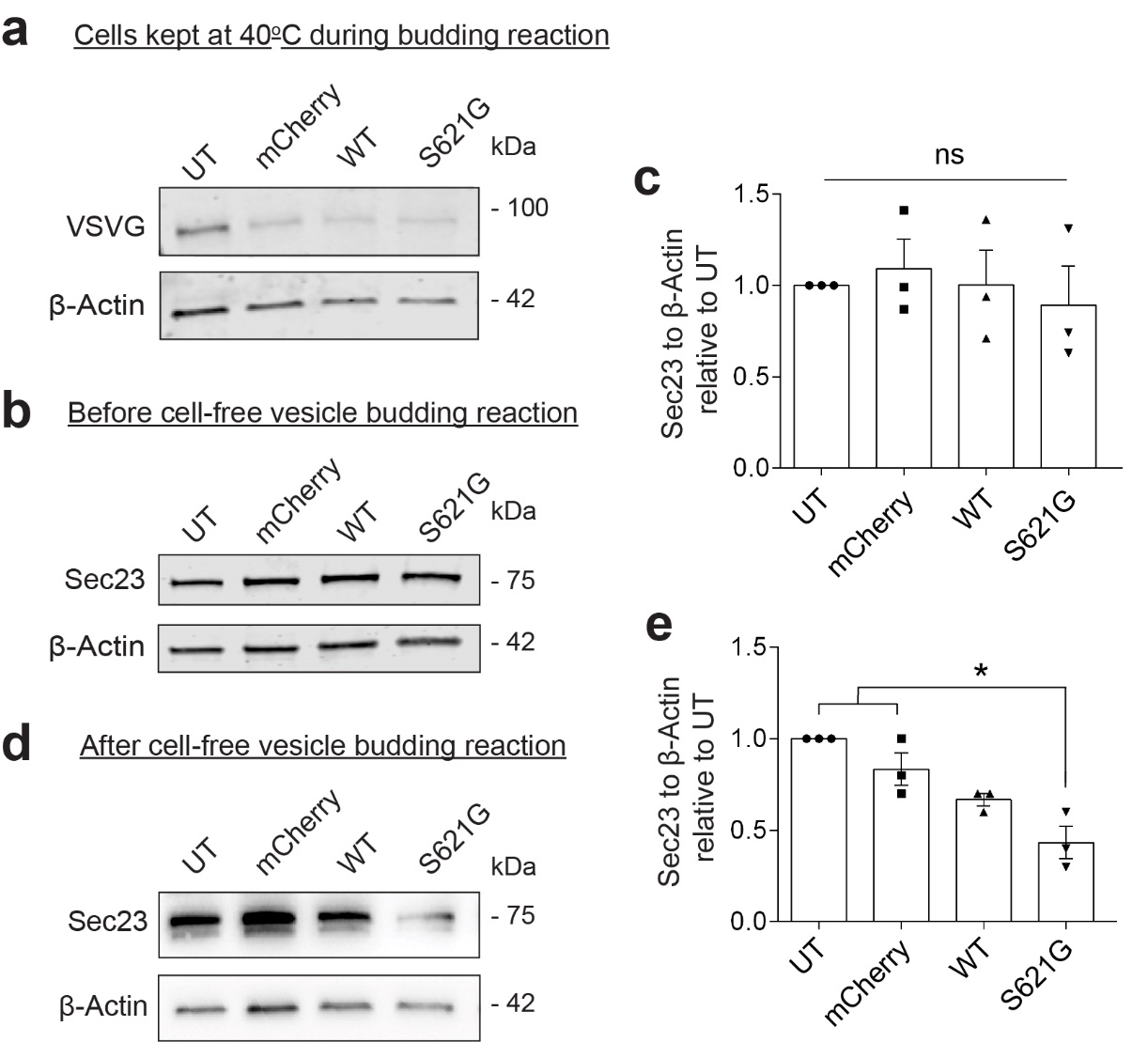


**Supplementary Figure 3 Whole cell analysis of Sec31-positive clusters in HEK293T cells expressing cyclin F^WT^ and cyclin F^S621G^.**

(a) Size distribution of diameters of Sec31-positive clusters from whole cell images acquired at 100x objective magnification using confocal laser microscopy. Untransfected (UT) cells and mCherry tagged empty vector were used as controls. Clusters up to 1000nm in diameter were resolved in 100 nm bins.

(**b**) Western blotting of Sec31A relative to β-actin in lysates from UT or cells expressing mCherry, cyclin F^WT^ or cyclin F^S621G^. β-actin as loading control.

(**c**) Sec31A relative to β-actin. Mean ± SEM; One-way ANOVA, post-hoc Tukey test; ns, non-significant. There were no significant differences in total Sec31A expression between the groups.


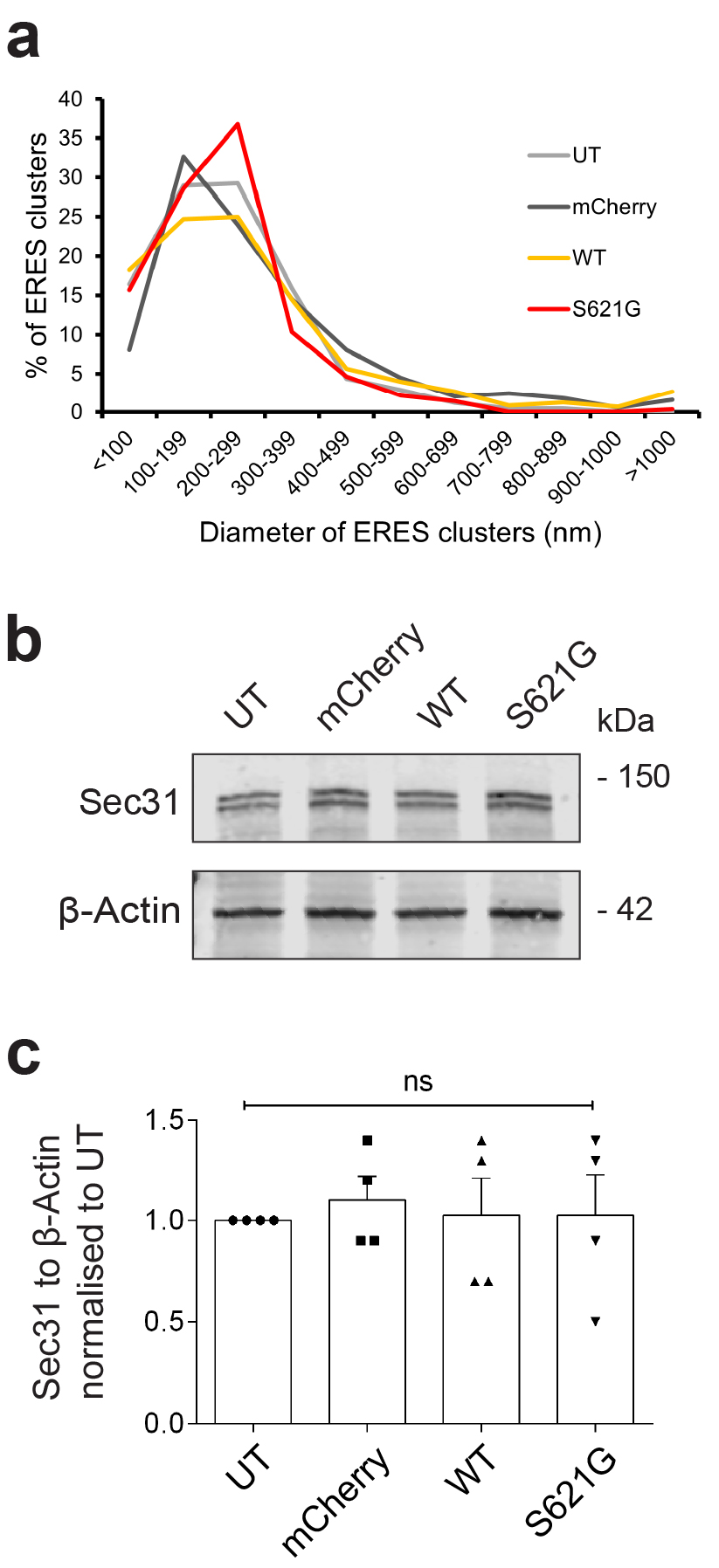


Supplementary Figure 4 ALS/FTD-associated cyclin F variants and **cyclin F^WT^ accumulate in the ER to a similar degree.**

(**a**) Western blotting of ER-rich containing cellular fractions from cyclin F^WT^, cyclin F^S621G^, cyclin F^S195R^ expressing cells, for calnexin and GADPDH as ER and cytoplasmic markers, respectively, and cyclin F.

(**b**) Quantification of western blots in (**c**), n=2. No significant differences were detected between the levels of cyclin F variants and cyclin F^WT^ in the ER fraction**.**

(**c**) Immunocytochemistry for calnexin as an ER marker in cells expressing cyclin F^WT^ or cyclin F^S621G^ for 48 hr.

(**d**) The degree of co-localisation of calnexin with cyclin F from the images in (**a**) was quantified using Mander’s coefficient, ns non-significant.


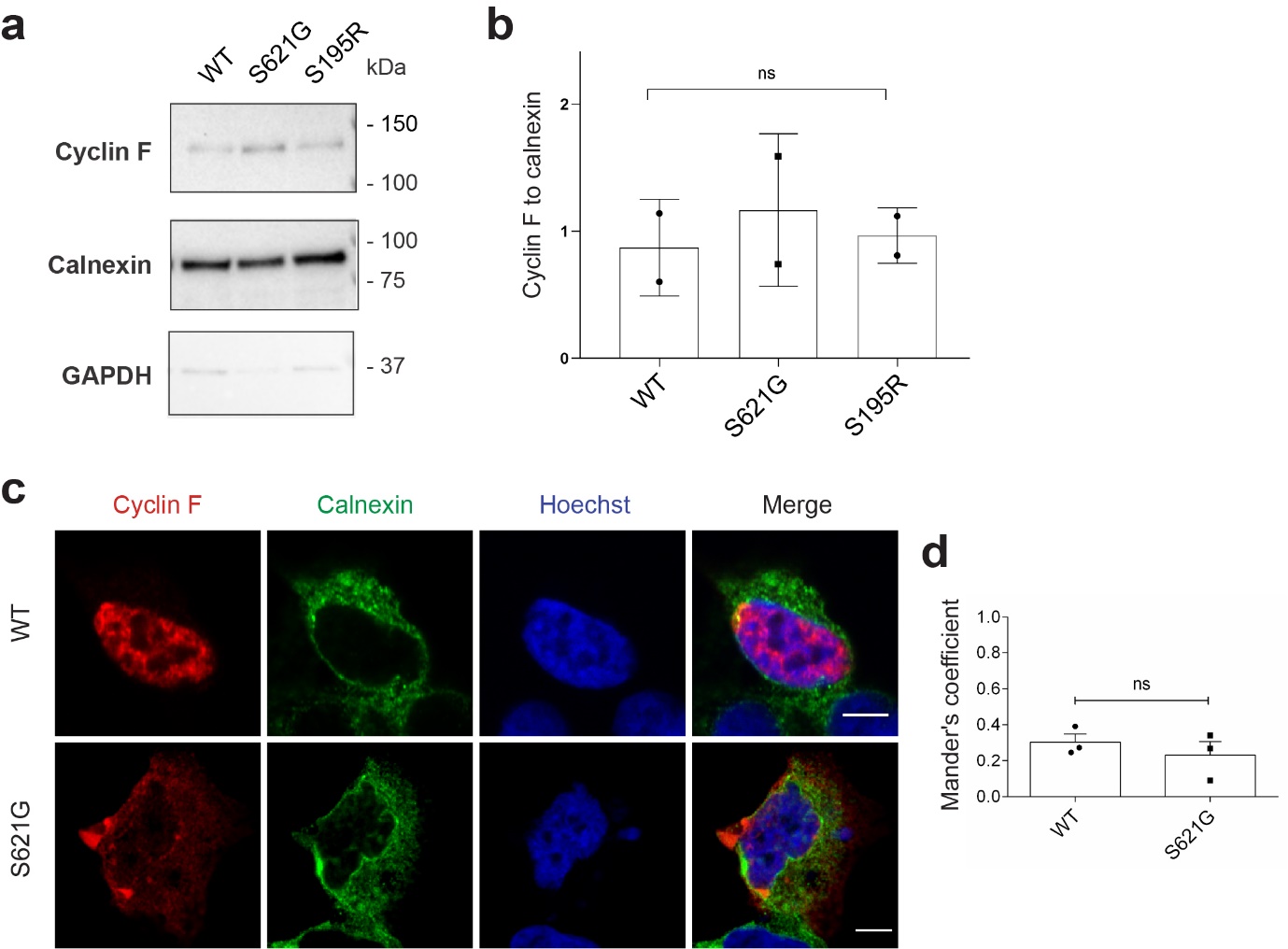


Supplementary Figure 5 **ALS/FTD variant cyclin F^S621G^ induces nuclear immunoreactivity for XBP-1 in mouse cortical primary neurons.**

Fluorescent confocal microscopy images, following immunocytochemistry for XBP-1 and Hoechst staining, of primary neurons expressing mCherry-tagged cyclin F^S621G^. Nuclear localisation of XBP-1 was detected in primary neurons expressing cyclin F^S621G^.


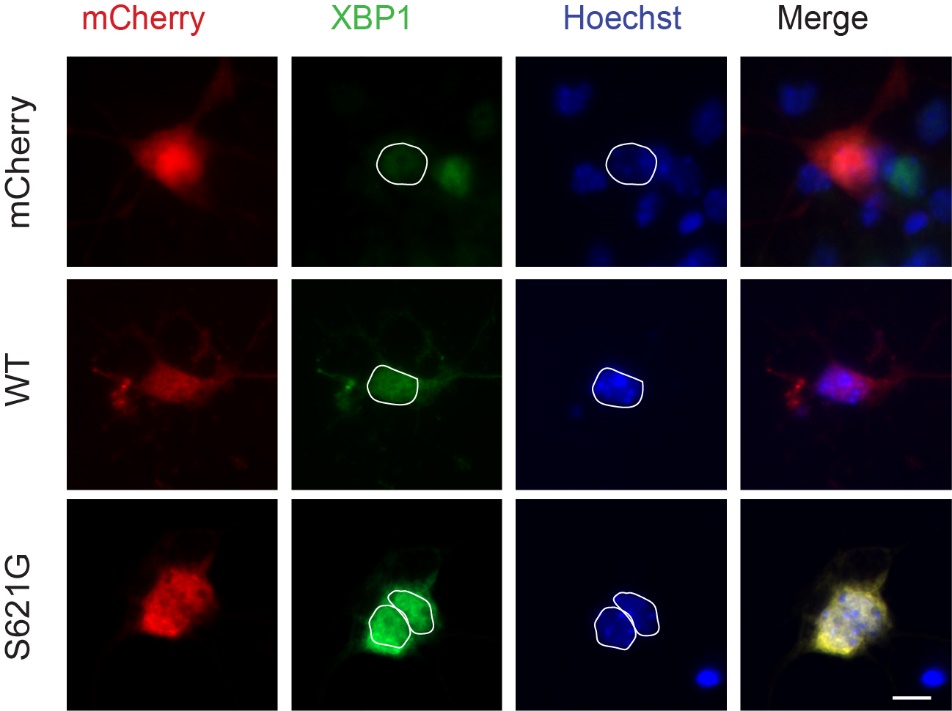


Supplementary Figure 6 **ALS/FTD variant cyclin F^S621G^ induces Golgi fragmentation in HEK293T cells.**

**(a**) Fluorescent confocal microscopy images of mCherry only or cyclin F, following immunocytochemistry for GM130 and Hoechst staining, of HEK293T cells expressing mCherry or mCherry-tagged cyclin F. Arrows: Golgi fragments. Scale bar = 10 *µ*m

(**b**) The proportion of cells with fragmented Golgi in **(a)** was quantified.

The graph represents mean ± SEM, n = 50+ cells per group were analysed from 3 independent experiments, one-way ANOVA followed by a post-hoc Tukey test for multiple comparisons, ***p*<0.01 and ****p*<0.001 *vs* UT and mCherry, ^##^*p*<0.01 *vs* cells expressing cyclin F^WT^.


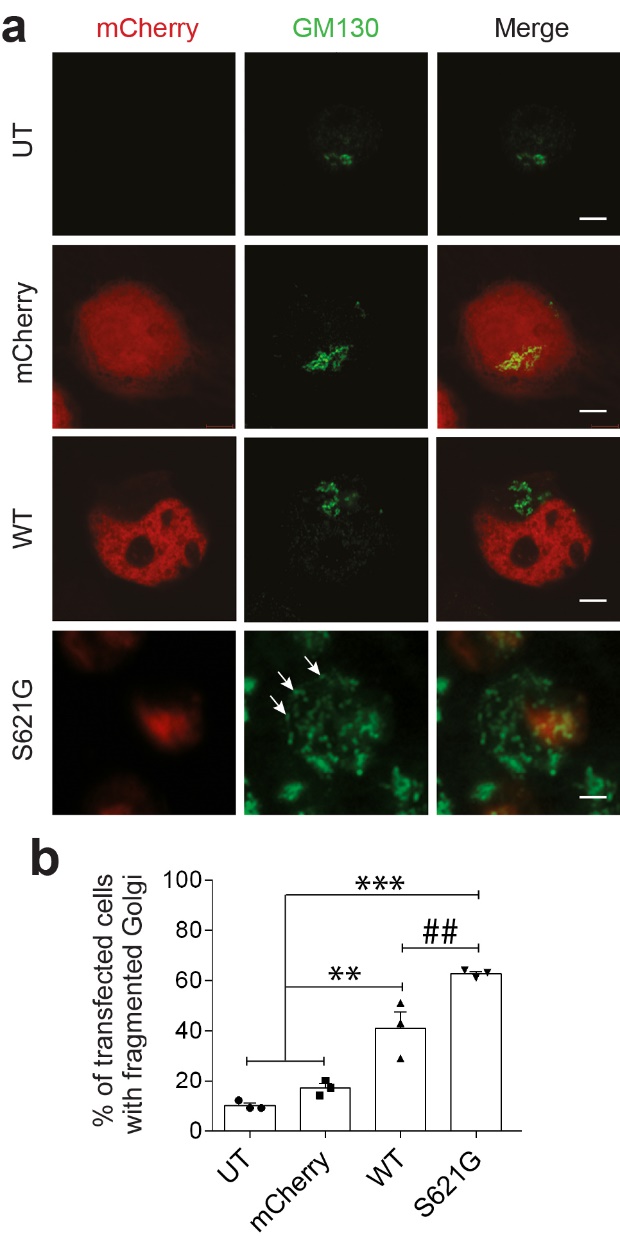


Supplementary Figure 7 **ALS/FTD variant cyclin F^S621G^ induces Golgi fragmentation in SH-SY5Y cells co-expressing cyclin F and VSVG^ts045^ and incubated at 40^o^C overnight (see Figure 1**).

(**a**) The proportion of cells with fragmented Golgi in **Figure 1** was quantified.

(**b**) The area covered by Golgi fragments was measured in each cell.

(**a, b**) The graphs represents mean ± SEM, n = 30+ cells per group were analysed from 3 independent experiments, one-way ANOVA followed by a post-hoc Tukey test for multiple comparisons, ns, non-significant (WT vs mCherry), **p*<0.05, ***p*<0.01 and ****p*<0.001 *vs* mCherry only; ^#^*p*<0.05, ^###^*p*<0.001 *vs* cells expressing cyclin F^WT^.


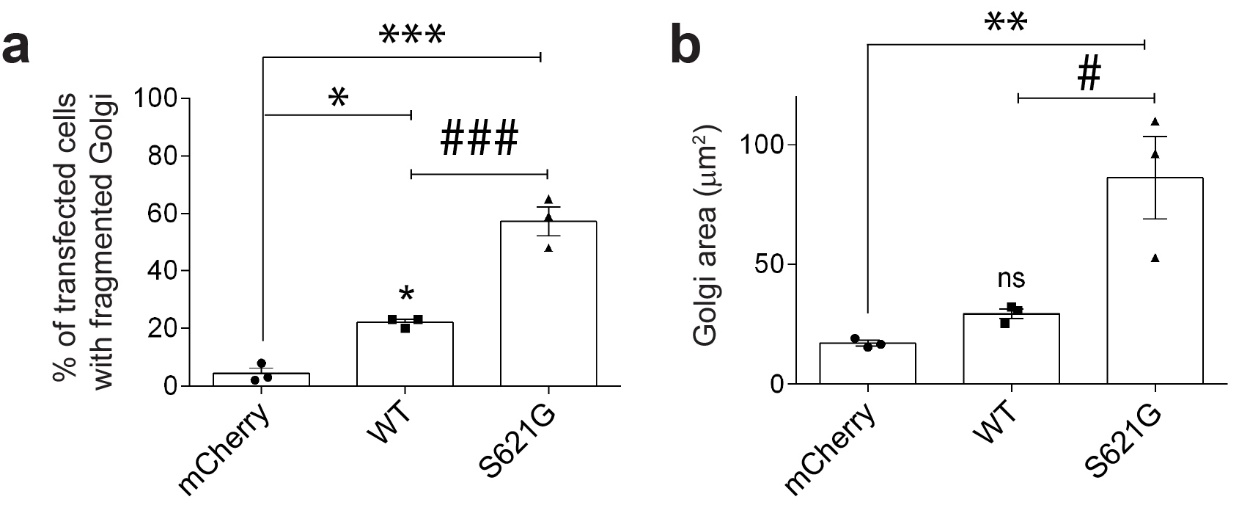


Supplementary Figure 8 **ALS/FTD variant cyclin F^S195R^ induces Golgi fragmentation and ER stress in SHSY5Y cells and mouse cortical primary neurons.**

**(a**) Fluorescent confocal microscopy images, following immunocytochemistry for GM130 and Hoechst staining, of SHSY5Y cells expressing mCherry-tagged cyclin F^S195R^. Arrows: Golgi fragments. Scale bar = 10 *µ*m.

(**b**) The proportion of cells with fragmented Golgi in **(a)** was quantified; images of untransfected cells (UT), mCherry only, cyclin F^WT^ shown in **Figure 6**. Graph represents mean ± SEM, n=50+ cells/group, Symbols represent 3 independent experiments, one-way ANOVA followed by a post-hoc Tukey test, ***p*<0.01 and ****p*<0.001 *vs* UT and mCherry only, ^##^*p*<0.01, *vs* cells expressing cyclin F^WT^

(**c**) The area covered by fragmented Golgi was quantified per cell using the images in (**a**); images of untransfected cells (UT), mCherry only, cyclin F^WT^ shown in **Figure 6**. Mean ± SEM. Symbols represent 3 independent experiments, n = 50+ cells per group one-way ANOVA followed by a post-hoc Tukey test for multiple comparisons; ns, non-significant (WT vs UT and mCherry), ****p*<0.001 *vs* UT and mCherry; ^##^*p*<0.01 *vs* cells expressing cyclin F^WT^,

(**d**) Fluorescent confocal microscopy images of following immunocytochemistry for GM130 and Hoechst staining in mouse primary cortical neurons expressing cyclin F^S195R^. Arrows: fragmented Golgi. Dashed white line delimits the outline of the neuron. Scale bar = 5 *µ*m.

(**e**) The proportion of primary neurons with fragmented Golgi in (**c**) was quantified; images of untransfected cells (UT), mCherry only, cyclin F^WT^ shown in **Figure 6**. Apoptotic neurons displaying a condensed nucleus were excluded from analysis. Mean ± SEM. Symbols represent 3 independent experiments, n = 10-30 neurons per group; one-way ANOVA followed by a post-hoc Tukey test for multiple comparisons; ****p*<0.001 *vs* UT and mCherry; and ^##^*p*<0.01 *vs* cells expressing cyclin F^WT^.

(**f**) Fluorescence microscopy of mouse primary cortical neurons expressing mCherry-tagged cyclin F^S195R^, following immunocytochemistry for CHOP and Hoechst. Nuclei: white outline. Arrows: nuclear CHOP immunoreactivity. Scale bar=10 *μ*m.

(**g**) Proportion of neurons with nuclear CHOP in (**f**): images of untransfected cells (UT), mCherry only, cyclin F^WT^ shown in **Figure 5**. Neurons undergoing apoptosis (with condensed nuclei) were excluded. Mean ± SEM; n = 10-30 neurons/group; one-way ANOVA, post-hoc Tukey test. ns, non-significant (WT vs mCherry), ****p*<0.001 *vs* untransfected cells (UT) and mCherry only; ^##^*p*<0.01 *vs* cells expressing cyclin F^WT^.

(**h**) Representative fluorescent microscopy images of mouse cortical primary neurons expressing cyclin F^S195R^, stained with Hoechst. Condensed nuclei (arrows), indicative of neuronal death, are present in cells expressing mutant cyclin F. Scale bar = 10 *µ*m.

(**i**) The proportion of cells with condensed, fragmented nuclei in (**h**) was quantified, mCherry and cyclin F^WT^ shown in **Figure** 7. Mean ± SEM. Symbols represent n=3-4 independent experiments, one-way ANOVA, followed by post-hoc Tukey test for multiple comparisons, **p*<0.05, ****p*<0.001 compared to untransfected cells (UT) and mCherry only; ^#^*p*<0.05 compared to cyclin F^WT^.


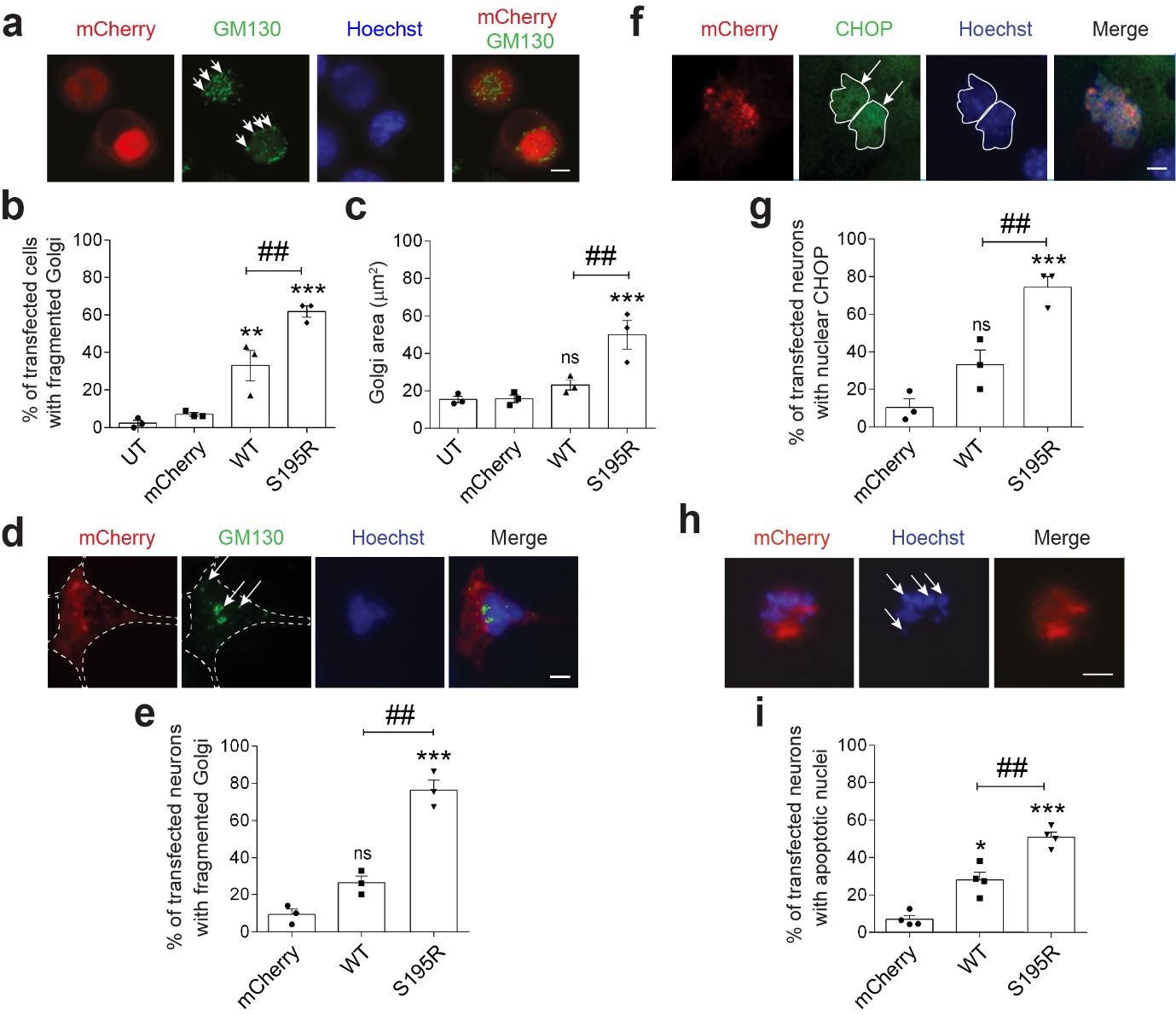

Supplement: Supplementary file 1 — Supplementary Figures. [file 41598_2023_46802_MOESM1_ESM.docx]
